# An MOF‐Enhanced Anti‐Fouling Immunoprobe Platform for Efficient Direct Screening of Pancreatic Cancer

**DOI:** 10.1002/advs.202503619

**Published:** 2025-07-01

**Authors:** Wenjuan Yang, Shujuan Mao, Hao Zhu, Handong Yao, Wei Jin, Lingyan Feng, Xinghua Gao, Xuefeng Wang, Wei Chen, Min Tu, Yuan Zhang

**Affiliations:** ^1^ Materials Genome Institute Shanghai Engineering Research Center for Integrated Circuits and Advanced Display Materials Shanghai University Shanghai 200444 China; ^2^ State Key Laboratory of Transducer Technology Shanghai Institute of Microsystem and Information Technology Chinese Academy of Sciences Shanghai 200050 China; ^3^ Department of Emergency Tongji Hospital Tongji University School of Medicine Shanghai 200065 China

**Keywords:** anti‐fouling immunoprobe, cancer biomarker detection, early diagnosis, machine learning, metal‐organic framework

## Abstract

Monitoring biomarkers offers insights for early disease (e.g., cancer, chronic diseases) screening, treatment guidance and response evaluation. To tackle challenges in precise biomarker detection in complex blood samples, we propose a metal‐organic framework (MOF)‐enhanced anti‐fouling immunoprobe (MAIP). This sensing platform integrates several key features, including an anti‐fouling surface, fully oriented antibody immobilization, and an enhanced electron transfer layer. This platform demonstrates remarkable sensitivity and specificity, capable of detecting trace concentrations of cancer markers, ranging from pg/mL to ng/mL. Comprehensive analysis of clinical serum samples, combined with machine learning (ML) evaluations, underscores the platform's reliability and effectiveness in cancer biomarker detection. Using a combination of three biomarkers, the algorithm demonstrated satisfactory classification performance with an accuracy of 100% on the test set across 64 serum samples. Moreover, when evaluated using 10‐fold cross‐validation, the optimized model maintained a robust accuracy of 0.955 ± 0.09, reinforcing its strong stability and good generalization ability across different data partitions. The MAIP‐based platform developed here offers swift analysis, high accuracy, and a simplified testing process. This makes it a promising approach for directly profiling cancer markers from blood, thereby facilitating timely and effective patient care.

## Graphical abstract

This work introduces an electrochemical sensing method that employs an innovative platform based on metal‐organic framework (MOF) for detecting biomarkers. The platform integrates an antifouling surface, oriented antibody immobilization, and an enhanced electron transfer layer. When combined with machine learning, it validates the platform's effectiveness in differentiating cancer cells from normal with high accuracy. This approach offers a reliable tool for personalized diagnostics and timely treatment.

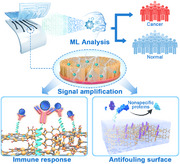

## 1 Introduction

In vitro diagnosis (IVD) has emerged as a pivotal technology among clinical testing methodologies, enabling the analysis of disease‐related biomarkers within blood, sweat, urine, or tissues to identify clinical diagnostic insights.^[^ ^1^, ^2^ ^]^ The development of highly sensitive and specific biosensors is crucial for the early diagnosis and effective treatment of many diseases. The sensitivity of most sensing technologies, though adequate for diagnosing diseases with obvious symptoms, struggles to detect trace‐level biomarkers that are crucial for identifying early‐stage infections, cancers, and neurological disorders. For instance, exosomes are widely used as liquid biopsy biomarkers for cancer screening and diagnosis due to their advantages of good stability, high sensitivity, and specificity.^[^ ^3^, ^4^ ^]^ However, the concentration of marker proteins carried on exosomes is often as low as ng/mL, which requires highly sensitive sensors.^[^ ^5^, ^6^, ^7^ ^]^

Affinity‐based electrochemical biosensors, leveraging specific molecular recognition elements (e.g., antibodies, aptamers, and enzymes), have emerged as one of the most promising technologies in biomarker analysis.^[^ ^8^, ^9^ ^]^ These electrochemical biosensors are capable of detecting trace amounts of biomarkers at concentrations below ng/mL, making them highly valuable in clinical medicine.^[^ ^10^, ^11^, ^12^ ^]^ However, when tasked with detecting trace biomarkers in complex body fluid environments, these biosensors encounter several challenges, such as non‐specific binding and background noise, which limit their accuracy and reliability in clinical applications.^[^ ^13^ ^]^ Optimizing blood sample pretreatment can mitigate these issues, yet it remains a time‐consuming process that is prone to introducing deviations and inaccuracies in the analytical results.^[^ ^14^, ^15^ ^]^ In immunosensing, oriented antibody immobilization is a widely accepted principle in biosensor design, which is crucial for improving the sensitivity, selectivity, and reliability of the sensor. Notably, remarkable progress has been made in metal organic frameworks (MOF) mediated antibody immobilization strategies. For example, utilizing the fragment crystallizable (Fc) region of antibody to trigger the crystallization of zinc‐based MOF enables selective growth of antibody with the correct spatial orientation on MOF nanocrystals.^[^ ^16^, ^17^ ^]^ This MOF‐mediated antibody oriented‐immobilization approach offers simplicity, preserves antibody binding functionality, and exhibits broad applicability in biosensing, diagnostic imaging, and targeted drug delivery.

**Figure 1:**
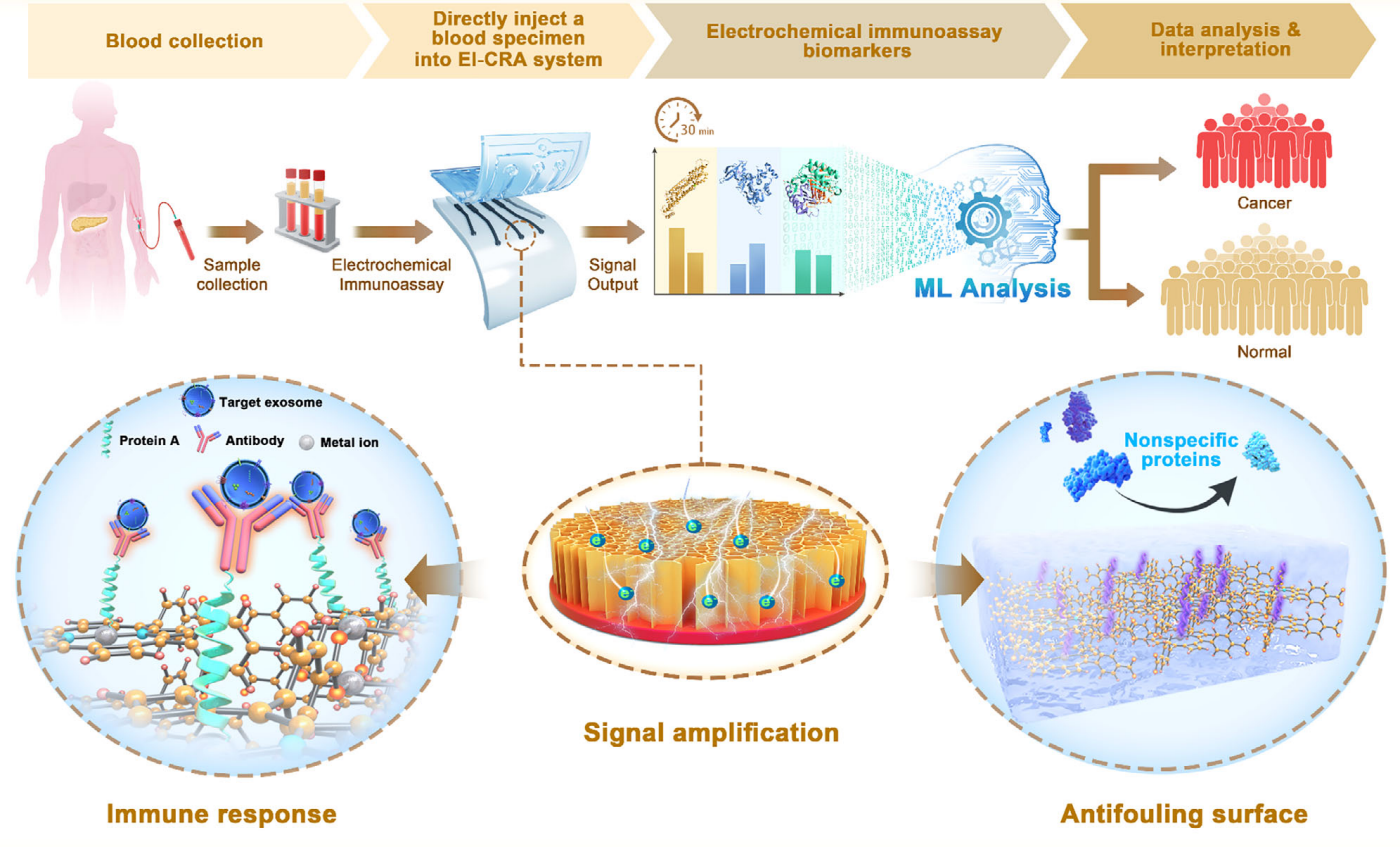
Schematic illustrating the cancer risk analysis process using the developed three‐channel electrochemical sensing platform based on MAIP. EI‐CRA refers to electrochemical immuno‐cancer risk analysis.

Herein, we report a MOF‐enhanced anti‐fouling immunoprobe (MAIP) and develop a three‐channel electrochemical sensing platform. This platform demonstrates promising for the direct detection of cancer markers from blood samples and has been validated for use in pancreatic cancer diagnosis. The developed MAIP is designed by oriented immobilization of recognition antibody on a nanoporous electron transfer layer, combined with anti‐fouling treatment of the sensing interface (**Figure** **1**). We employed a nanostructured electrically conductive MOF layer to enhance electron transfer, achieving a desired detection sensitivity. Recognition antibodies are immobilized on the MOF layer to ensure the proper spatial configuration for the specific capture of target proteins. An anti‐fouling surface constructed with peptide molecules ensures detection sensitivity in a complex serum matrix.

Benefiting from the high accuracy and efficiency of the developed platform, we propose an electrochemical immuno‐cancer risk analysis (EI‐CRA) strategy for pancreatic cancer screening. Three cancer‐correlated biomarkers (epidermal growth factor receptor (EGFR), one pancreatic cancer marker (glypican‐1, GPC1) and macrophage migration inhibitory factor (MIF)) can be detected by the developed MAIP‐based platform with a detection limit as low as 5 pg mL^−1^. Machine learning (ML) algorithms, including Random Forest (RF) and Neural Network (NN) were employed to analyze the sensing results from clinical samples. The clinical sample data analysis reveals that combining the three biomarkers results in both algorithms (RF and NN) achieving 100% accuracy. The proposed MAIP‐based sensing platform overcomes the limitations of biosensor technology in achieving highly sensitive and specific detection in complex biological fluids, notably blood samples, paving the way for the development of more accurate diagnostic tools. This breakthrough opens new avenues for precision medicine and enhances our ability to detect and manage diseases at earlier stages.

## 2 Results and Discussion

### 2.1 Sensing Materials Synthesis and Characterization

**Figure 2:**
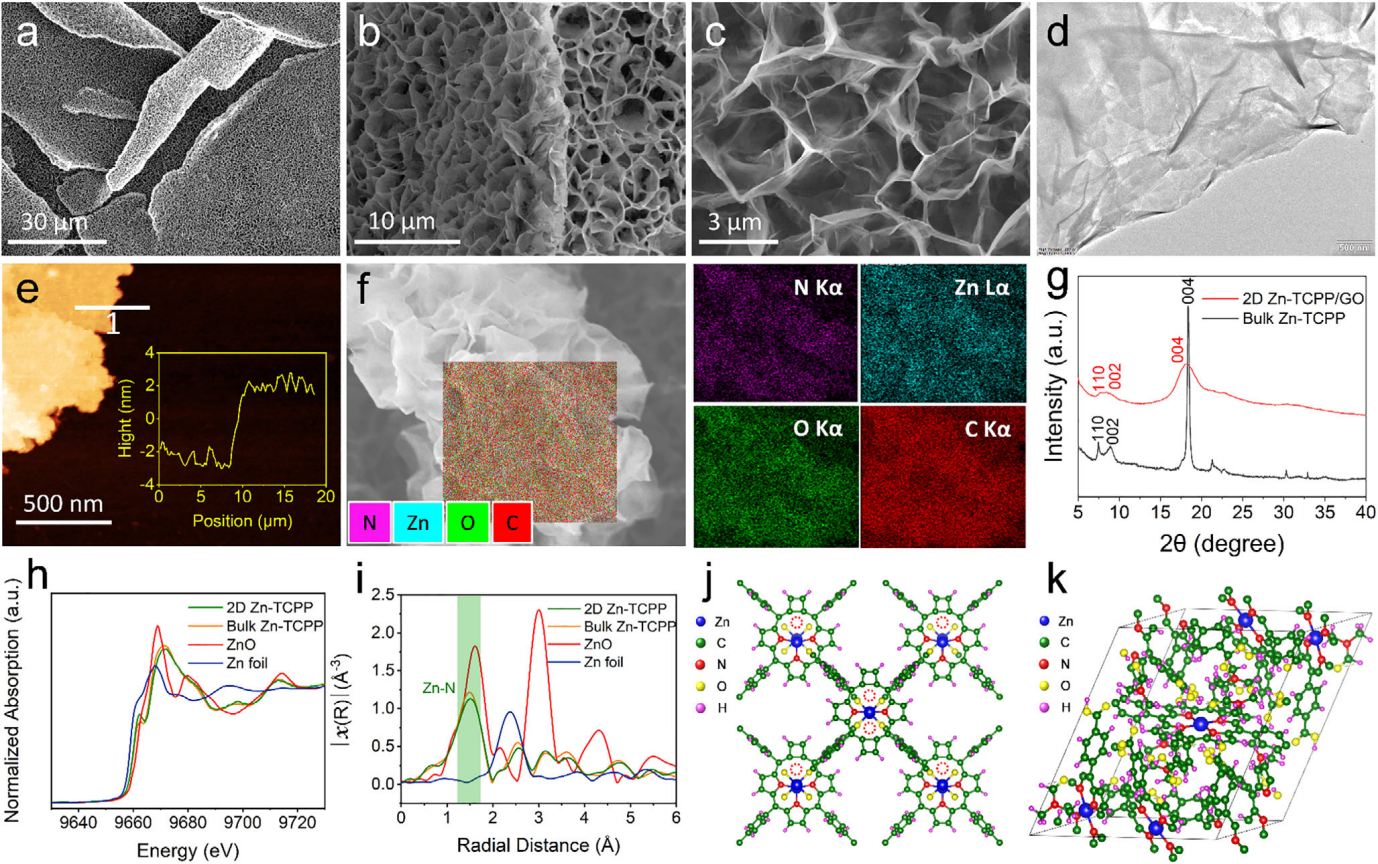
a–c) SEM images of 2D Zn‐TCPP/GO composite. d–f) TEM, AFM and EDS characterization results of ultra‐thin Zn‐TCPP nanosheets, respectively. g) XRD patterns of 2D Zn‐TCPP/GO composite. h) Zn K‐edge XANES spectra of 2D Zn‐TCPP, bulk Zn‐TCPP, ZnO and Zn foil. i) EXAFS spectra of 2D Zn‐TCPP, bulk Zn‐TCPP, ZnO and Zn foil. j,k) Structure models of 2D Zn‐TCPP and bulk Zn‐TCPP. ZnO and Zn foil are standard reference samples.

In this study, 2D MOF of Zn(II) tetrakis(4‐carboxyphenyl)porphyrin (2D Zn‐TCPP) owing large surface area and abundant accessible active sites for robust interactions with biomolecules, was employed as sensing materials. Zn‐TCPP nanosheets offer binding sites for the oriented immobilization recognition antibodies as well as the modification of antifouling polypeptide molecules (Figure S1, Supporting Information). Zn‐TCPP nanosheets were in situ grown on the conductive graphene oxide (GO) surface for providing efficient electron transfer channels. The scanning electron microscopy (SEM) images confirm the uniform growth of 2D Zn‐TCPP on the GO surface (**Figure** **2**a–c). The orientated arrangement of nanosheets produces a porous network microstructure on the surface of GO with porosity from sub‐microns to a few microns. Such a structure significantly contributes to the improvement of its electrochemical properties. The superiority of the composite structure in electrochemical sensing applications is evidenced by the observation that the current signal generated solely by 2D Zn‐TCPP is considerably weaker compared to that of the 2D Zn‐TCPP/GO composite (Figure S2, Supporting Information). Additionally, the 2D Zn‐TCPP/GO composite also exhibits excellent stability in terms of morphology, chemical structure, and electrochemical performance, as can be observed from Figures S3–S5, Supporting Information.

The ultrathin structural characteristics of the 2D Zn‐TCPP nanosheets can be clearly observed from the transmission electron microscopy (TEM) image (Figure 2d). The atomic force microscopy (AFM) result reveals the thickness of the prepared nanosheet is 4.2 ± 0.9 nm (Figure 2e). It suggests the number of layers is about 5 ± 1, according to the theoretical interlayer distance of 0.93 nm for 2D Zn‐TCPP.^[^ ^18^ ^]^ The energy dispersive X‐ray spectroscopy (EDS) analysis shown in Figure 2f indicates that the four elements (N, Zn, C and O) are evenly distributed in the probed area. Figure 2g displays the XRD patterns of both 2D Zn‐TCPP/GO and bulk Zn‐TCPP, revealing the formation of the MOF structure in the 2D Zn‐TCPP/GO sample through the presence of the same characteristic peaks. Similar results were also obtained from the Fourier transform infrared spectroscopy (FT‐IR) results (Figure S6, Supporting information). The characterization results indicate that the ultra‐thin Zn‐TCPP nanosheets have been successfully grown on the conductive GO substrate.

Synchrotron X‐ray absorption fine structure (XAFS) spectroscopy, including X‐ray absorption near‐edge structure (XANES) and extended X‐ray absorption fine structure (EXAFS), can provide valuable information on the coordination environment and chemical state of the absorbing atom with high sensitivity. The normalized XANES profiles of Zn K‐edge revealed that both 2D Zn‐TCPP and bulk Zn‐TCPP exhibit near‐edge absorption energies nearly identical to those of ZnO, indicating that the Zn sites exist as Zn (II) species (Figure 2h). Figure 2i displays the Zn K‐edge EXAFS spectra of 2D Zn‐TCPP and bulk Zn‐TCPP, exhibiting a characteristic peak of Zn‐N coordination bond in both samples.^[^ ^19^, ^20^ ^]^ The corresponding fitting results of Zn K‐edge EXAFS in k‐space and R‐space after the Fourier transform are shown in Figure S7a–d, Supporting Information. The structural parameters (coordination elements, bond lengths, coordination numbers, etc.) extracted from the EXAFS fitting (Table S1, Supporting Information) indicate that the coordination number (CN) of Zn‐N in the 2D Zn‐TCPP is 3.4 ± 0.2. In contrast, the CN of Zn‐N is 3.9 ± 0.2 in the bulk Zn‐TCPP sample. In bulk Zn‐TCPP, the coordination between metal centers and organic TCPP ligands results in the 3D channel structure of Zn‐TCPP with Zn‐N saturated tetra‐coordination. These results suggest that the Zn atoms in bulk Zn‐TCPP are coordinated with nearly four nitrogen atoms, whereas the 2D Zn‐TCPP sample contains a number of coordinated open unsaturated Zn sites. The proposed structure models for 2D Zn‐TCPP and bulk Zn‐TCPP are presented in Figure 2j,k. In 2D Zn‐TCPP, each TCPP ligand is connected by four Zn paddle‐wheel metal nodes Zn_2_(COO)_4_ to form an ultrathin layered structure. The XAFS calculation reveals that the unsaturated 2‐coordinate Zn and 3‐coordinate Zn models are dominant in 2D Zn‐TCPP. The open unsaturated metal ion sites in ultra‐thin Zn‐TCPP nanosheets could be used for the oriented immobilization of antibody and antifouling treatment. Further density of states (DOS) calculations reveals that 2D Zn‐TCPP has a significantly narrower band gap (≈0.444 eV) compared to bulk Zn‐TCPP, which has a band gap of ≈1.465 eV (Figure S8a,b, Supporting Information). We also measured the electrochemical output signals of 2D Zn‐TCPP and bulk Zn‐TCPP using the differential pulse voltammetry (DPV) approach (Figure S8c, Supporting Information). The average current output signal of 2D Zn‐TCPP/GO modified electrode is about 236.5 µA, nearly three times higher than that of bulk Zn‐TCPP/GO deposited electrode. In addition, the 2D Zn‐TCPP/GO modified electrode also demonstrates excellent batch‐to‐batch reproducibility and long‐term storage stability (Figure S9, Supporting Information). Therefore, 2D Zn‐TCPP with unsaturated metal ion sites facilitates the construction of high‐performance electrochemical immunosensors.

### 2.2 Construction of Anti‐Fouling Sensing Interface

Biological fluids often contain a large number of coexisting interfering substances, which can lead to false positives or contamination of the electrode surface. Anti‐fouling capability is a pivotal characteristic in biosensor applications, directly affecting the sensor's accuracy, sensitivity, and reliability. Therefore, an anti‐fouling sensing interface was constructed on the 2D Zn‐TCPP/GO composite. High hydrophilicity and low net charge are the two key features to be considered for creating a proper anti‐fouling sensing interface. High hydrophilicity enables the sensing interface to form a stable hydration layer, repelling non‐specific adsorptions and preventing adhering contaminants.^[^ ^21^ ^]^ In addition, a sensing interface with a low net charge minimizes electrostatic interactions with biomolecules (e.g., proteins, cells), thereby lowering the risk of non‐specific adsorption. Herein, zwitterionic peptides are employed to provide the sensing interface with anti‐fouling capability and further improve the specificity of developed sensors.

**Figure 3:**
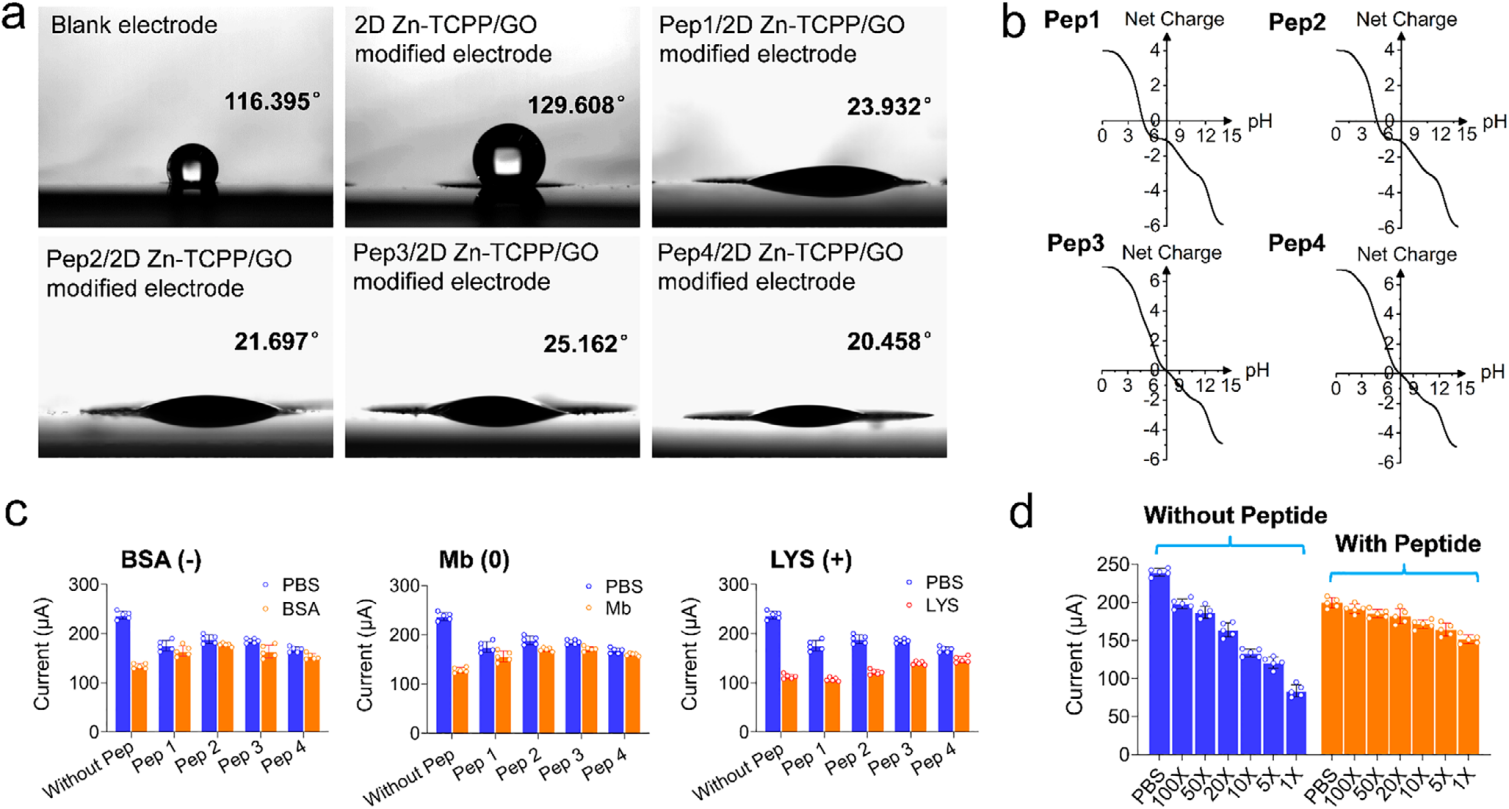
a) Static water contact angles of bare electrode, 2D Zn‐TCPP/GO modified electrode and peps/2D Zn‐TCPP/GO modified electrodes. b) Zeta potential testing results of the designed peptides in PBS environment (10 mM, pH = 7.4). c) Anti‐fouling performance of different peps/2D Zn‐TCPP/GO modified electrodes by using DPV in three typical protein environments. d) DPV current values of electrodes modified with and without pep 4 in blank PBS solution and real serum diluted with different multiples.

Peptide molecules with polar groups that facilitate hydrogen bonding with water molecules can provide excellent hydrophilicity. Meanwhile, the overall electroneutrality of the molecules can be achieved through the strategic arrangement of amino acid sequences. Here, two anti‐fouling peptides with different amino acid sequences are designed based on the principles of hydrophilicity and electroneutrality. Considering that most of the non‐specifically adsorbed proteins in biological fluids are negatively charged, two negatively charged peptide molecules are also designed for anti‐fouling purpose. The detailed structures of four designed peptide molecules as well as their theoretical isoelectric points and hydrophilicity index are shown in Figure S10, Supporting Information, Tables S2 and S3, Supporting Information, respectively. All the peptide molecules were designed to be terminated with cysteine, which contains a thiol group (‐SH) capable of coordinating with zinc ions in 2D Zn‐TCPP. X‐ray photoelectron spectroscopy (XPS) analysis results (Figure S11, Supporting Information) indicate that these peptide molecules can be successfully utilized for modifying the surface of 2D Zn‐TCPP through this coordination bond. Then, static water contact angle and zeta potential measurements are employed to characterize peptides modified electrode materials' wettability and surface charges, respectively. As displayed in **Figure** **3**a, both the bare electrode and 2D Zn‐TCPP/GO modified electrode surfaces are hydrophobic, whereas the attachment of peptides enhances hydrophilicity, likely due to the presence of amino (‐NH_2_) and carboxyl (‐COOH) groups in zwitterionic structures.^[^ ^22^ ^]^ The average zeta potential values of Pep 1 and Pep 2 are −15.7 and −22.2 mV, respectively, suggesting the negative surface charges (Figure 3b). which may have an anti‐fouling effect on most proteins that are negatively charged in physiological environments. The average zeta potential values of Pep 3 and Pep 4 are 0.674 and 0.723 mV, respectively. This indicates that the two peptides are almost electrically neutral under physiological pH conditions, which meet the design principle of electroneutrality of antifouling materials.

We then evaluated the anti‐fouling properties of different peptides by using DPV in three typical protein environments. Here, bovine serum albumin (BSA), myoglobin (Mb) and lysozyme (LYS, Egg White) with the isoelectric points of 4.70, 7.07 and 10.80, respectively, are used to simulate the negatively charged, neutral and positively charged protein environments. The attenuation percentage (η) of DPV currents in blank PBS solution and simulated protein solution is used to evaluate the anti‐fouling performances of different peptides. The testing results demonstrate that pep 1, 2 and 4 display high anti‐fouling performance in BSA environment (Figure 3c and Figures S12–S14, Supporting Information), while pep 3 and pep 4 exhibit excellent anti‐fouling capability in Mb environment. Pep 1, 2, and 3 show strong electrostatic attraction to the positively charged LYS protein, which reduces their antifouling ability. Through the comparison mentioned above, pep 4 maintained relatively high antifouling ability in three different protein environments. The pep 4 modified electrode also shows high anti‐fouling efficiency toward real serum, as evidenced by the minimum current signal fluctuations between tests with PBS solution and diluted serum samples (Figure 3d, Figure S15, Supporting Information). Therefore, pep 4 was employed on the 2D Zn‐TCPP/GO composite for the construction of anti‐fouling interface.

### 2.3 Design and Performance Characterization of MAIP‐Based Sensing Platform

**Figure 4:**
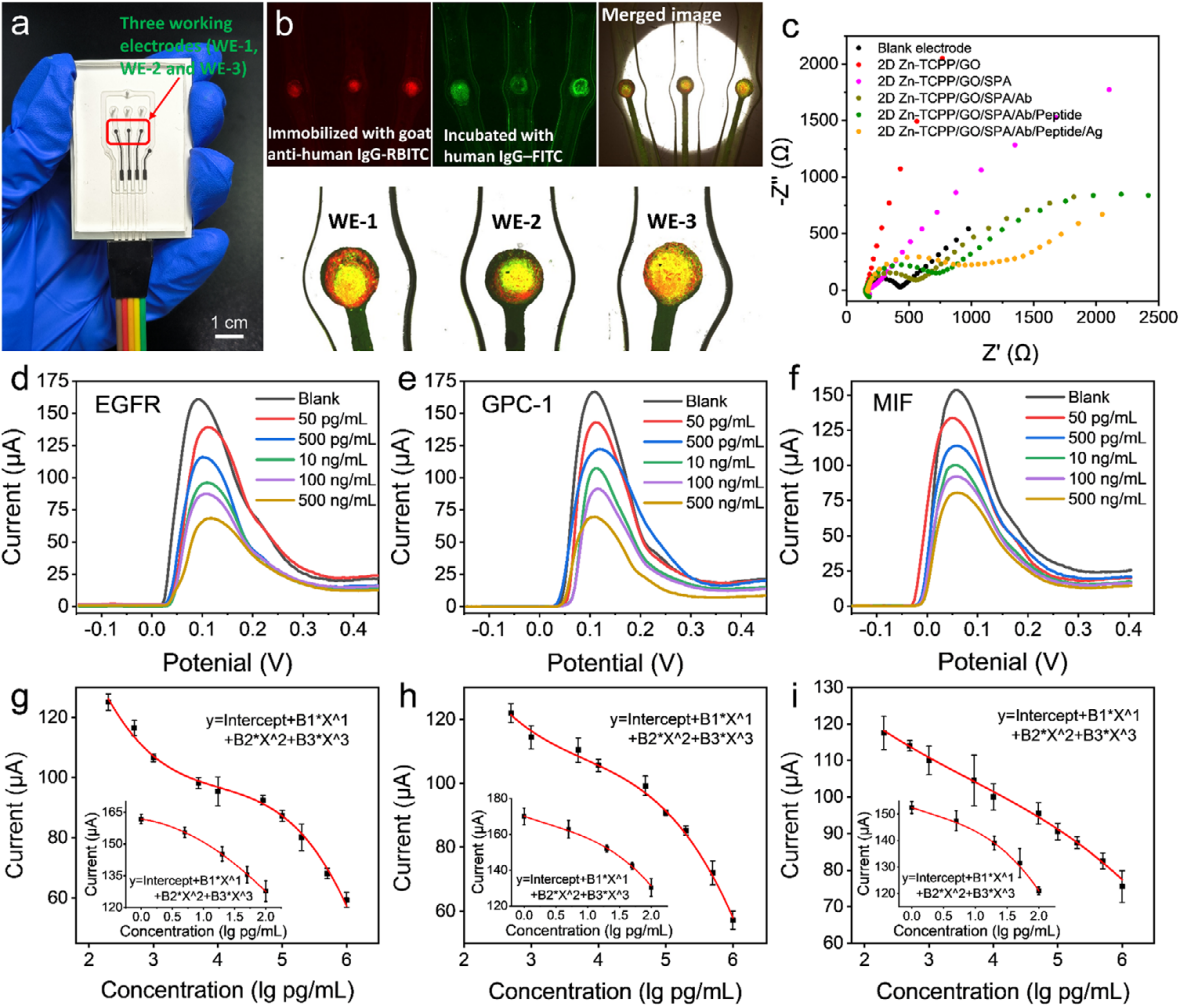
a) Configuration of three‐channel detection chip. b) Fluorescence characterization of immunoreaction process on the electrodes. c) Nyquist plots representing bio‐recognition events at the electrode surface. SPA: Protein A, Ab: goat anti‐human IgG, Ag: human IgG. d–f) DPV sensing responses of the developed MAIP‐based platform for EGFR, GPC‐1 and MIF at various concentrations. g‐i) Corresponding dose‐dependent responses toward EGFR, GPC‐1 and MIF.

Ultra‐thin Zn‐TCPP nanosheets with unsaturated metal sites enable oriented antibody‐immobilization and zwitterionic peptide modification, which can combine immunoassay and anti‐fouling treatment into a single unit. Further combined with a three‐channel detection chip (**Figure** **4**a), a MAIP‐based sensing platform is constructed. The confocal fluorescence microscopy observation of fluorescently labeled antibody and antigen demonstrates the efficient immobilization of antibody as well as immunoreaction (Figure 4b). After immobilized with goat anti‐human IgG‐RBITC, three clear red spots are presented in the working electrodes area, and then turn to green with the specific immune response. Electrochemical impedance spectroscopy (EIS) was used to analyze the interfacial properties of bio‐recognition events at the electrode surface. The corresponding Nyquist plots of impedance spectra are shown in Figure 4c, where Z′ represents the real part and ‐Z′′ describes the imaginary part of the complex impedance over the frequency range of 100 kHz–10 MHz with an AC amplitude of 10 mV. The bare electrode shows a semicircle in the high frequency region of the Nyquist plot. However, the plot of 2D Zn‐TCPP/GO modified electrode is almost a straight line, indicating the fast electron transfer on the electrode interface. After binding with protein A and goat anti‐human IgG, the semicircle radius increases gradually at high frequency. This can be attributed to the attachment of protein and antibody molecules on 2D Zn‐TCPP/GO, thus hindering electron transport. Subsequently, the anti‐fouling peptide modification produces an even larger semicircle, suggesting slow electron transfer due to the biomolecular blocking effect. The addition of human IgG further increases electron transfer resistance in the high‐frequency region, indicating the presence of a steric hindrance effect at the interface due to the formation of immuno‐complexes. We subsequently chose goat anti‐human IgG as the antibody and human IgG as the antigen to assess the sensing capability of our developed platform. After optimization on the amount of protein A and goat anti‐human IgG (Figures S16 and S17, Supporting Information), the developed MAIP‐based platform achieves a wide detection range from 5 pg mL^−1^ to 1000 ng mL^−1^ for human IgG (Figure S18, Supporting Information). We have also evaluated the SEM result and FT‐IR spectrum of the 2D Zn‐TCPP/GO modified electrode after the sensing test. As shown in Figures S19 and S20, Supporting Information, the Zn‐TCPP nanosheets exhibit excellent morphology and chemical stability, further confirming the robustness of the MOF‐based detection platform.

### 2.4 Tumor Markers Detection and Evaluation of the MAIP‐Based Platform for Pancreatic Cancer Screening

For the choice of biomarkers, we primarily focus on the following three cancer‐correlated biomarkers: EGFR, GPC1 and MIF. EGFR is known to be a membrane protein that plays a dominant role in tumorigenesis and development. Recent studies found that EGFR can be secreted from cells via the transport of vesicles and these EGFR‐containing exosomes regulate signaling pathways of endothelial cells and T cells.^[^ ^23^, ^24^ ^]^ GPC1 is a cell surface proteoglycan, specifically enriched on cancer‐cell‐derived exosomes, that has been proved to be overexpressed in breast and pancreatic cancer.^[^ ^25^ ^]^ It has been demonstrated that diagnostic approaches leveraging GPC1‐associated exosomes exhibit high sensitivity and specificity in differentiating pancreatic cancer from benign pancreatic lesions.^[^ ^26^ ^]^ MIF is a pleiotropic cytokine with crucial roles in cell proliferation, tumorigenesis and angiogenesis, and has been found to be highly expressed in pancreatic cancer derived exosomes.^[^ ^27^ ^]^ The developed MAIP‐based platform shows sensing responses to all three proposed biomarkers in a wide range from pg/mL to ng/mL, and presents the same cubic polynomial model (shown in Figure 4d–i and Tables S4–S6, Supporting Information). The high sensitivity could be attributed to the designed MAIP, providing efficient recognition of the target protein.

**Figure 5:**
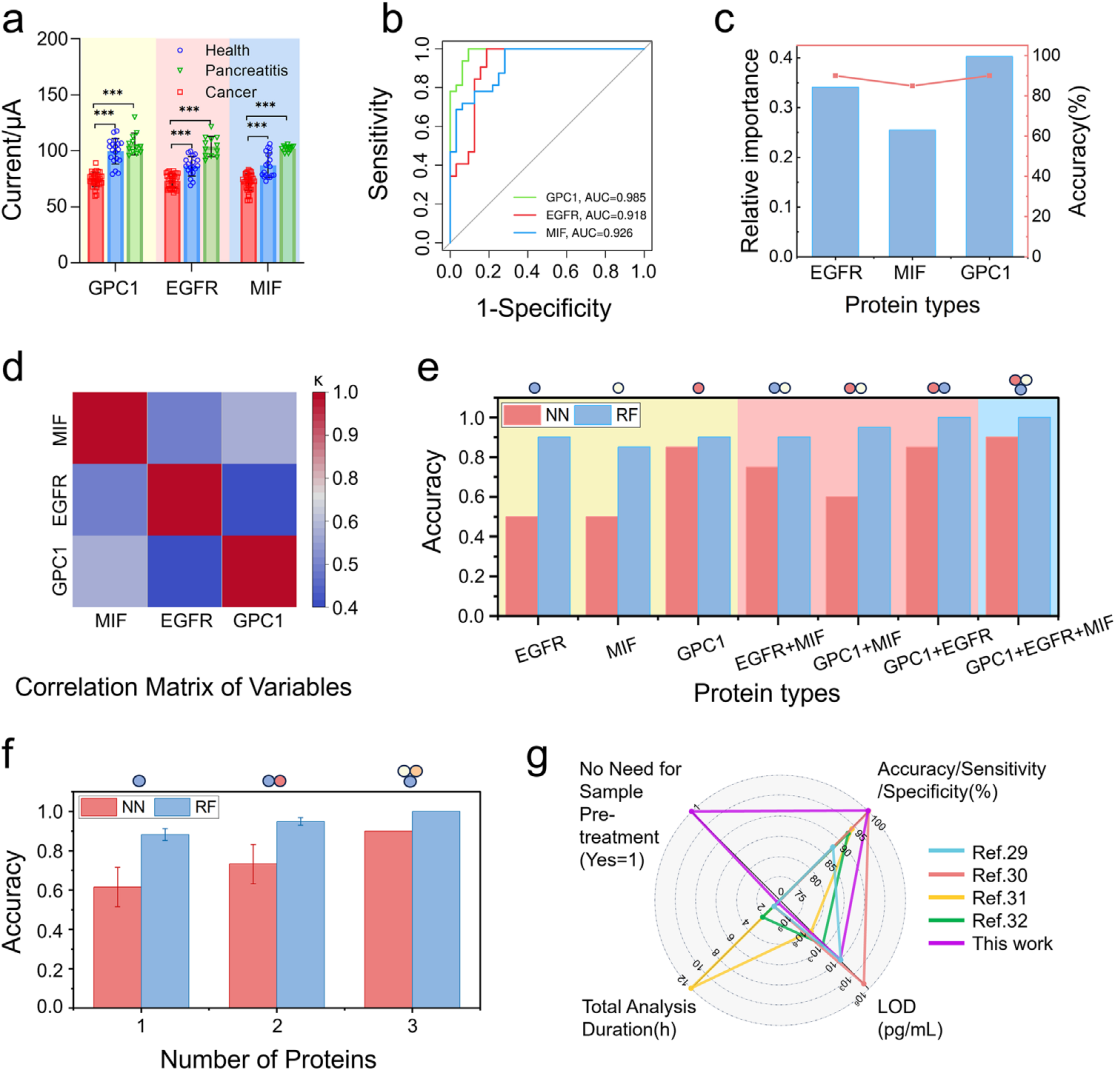
Profiling pancreatic cancer serum samples through the developed MAIP‐based platform. a) Analysis of serum samples from pancreatic cancer patients, patients with acute pancreatitis and healthy control cases. Each sample was tested independently 3–5 times. Differences with *** *p* < 0.0001 were considered statistically significant by Student's t test. b) ROC curve analysis results produced using data from clinical human serum assay. The collected set of sensing signals for each patient was then analyzed using ML to screen the patient for pancreatic cancer. c) Sensing characteristics of each biomarker provided by the random forest model. d) Pearson correlation coefficient matrix for biomarker combinations, with GPC1+EGFR having the lowest correlation (k = 0.40). e) Prediction accuracy of single biomarker and biomarker combinations in RF and NN models. f) Average accuracy predicted by both algorithms with different numbers of biomarkers. g) Radar chart summarizes the sensing performance of various detection methods or technologies.

A total 64 serum samples, including 32 from pancreatic cancer patients, 12 from patients with acute pancreatitis and 20 from healthy controls, were used to evaluate the practicability of the developed MAIP‐based platform. The clinical information collected from cancer patients and patients with acute pancreatitis is shown in Table S7, Supporting Information. Each serum sample was introduced into the three‐channel sensing platform, which had been pre‐conjugated with three different antibodies. After 30 min, the DPV peak currents were recorded as output sensing signals. Each sample provides three biomarkers sensing data points, with at least three tests for each sample. As shown in **Figure** **5**a, the clusters of obtained data indicate that there is a significant difference between pancreatic cancer group and both the acute pancreatitis patient and the healthy control. To further evaluate the diagnostic ability of the three markers, we plotted receiver operating characteristic (ROC) curves (as shown in Figure 5b). The area under the curve (AUC) values of the three markers are 0.985, 0.918, and 0.926 for GPC1, EGFR and MIF, respectively. These results indicate that GPC1 and MIF expression levels are more effective in distinguishing pancreatic cancer patients from those with acute pancreatitis and healthy controls. Notably, GPC1 demonstrates a superior discriminatory ability compared to MIF.

Since the employed student's t‐test has limitations in data processing, including restricted ability to handle multiple datasets, limited capacity in complex scenarios, and insufficient utilization of information. To overcome these limitations, we have developed a machine learning (ML) analysis method to explore the clinical potential of serum exosome marker profiling for diagnostic purposes. Two ML algorithms (NN and RF) are employed to analyze the relation of clinical outcome with the set of sensing signals from three different biomarkers. Both algorithms were trained on a subset of the clinical sample collection data set (consisting of 70% of the total data set, with n = 44 samples) under supervised learning conditions. Their performance was then tested by screening for pancreatic cancer in a blinded test set that comprised the remaining 30% of the total data set. The DPV current values from each biomarker testing were input into both algorithms, giving two different types of output: pancreatic cancer and normal.

The results of the ML analysis are shown in Figure 5c–f, and the relative importance and accuracy of each biomarker to the decision‐making outcome of the disease (from RF) is shown in Figure 5c. GPC1 showed relatively high importance (0.403) in the RF model, and its prediction accuracy was 90%. It is worth noting that the relative importance of EGFR was 0.329, which is higher than that of MIF. The prediction accuracy of EGFR is also higher than that of MIF, with EGFR achieving an accuracy of 90% and MIF achieving 85%. We also performed the SHapley Additive exPlanations (SHAP) value analysis for the contribution of three biomarkers combined in the RF model (Figure S21, Supporting Information). Through the analysis of SHAP values, we can quantify the contribution of each biomarker to the model's predictive outcomes, thereby identifying which biomarkers have a greater impact on the model's predictions. The results indicate that GPC1 exhibits a wider range of SHAP values, suggesting its more significant influence on the model's output. Although GPC1 had 90% accuracy in the RF model, its false positive rate was high in the NN model. The analysis results of single markers in the two algorithms differed greatly, and to achieve better accuracy in both models, we used a multi‐marker analysis method. As shown in Figure 5d, the Pearson correlation coefficient matrix for biomarker combinations indicates that the combination of GPC1 and EGFR has the lowest correlation (K = 0.40). Selecting biomarker combinations with lower correlation coefficients and using their sensing signals as inputs for ML can help achieve better results.^[^ ^28^ ^]^ The GPC1+EGFR combination achieved the highest prediction accuracy in both NN and RF models, with accuracies of 0.85 and 1.00, respectively. Furthermore, when three biomarker signals were combined, the prediction accuracy rates of the NN and RF models reached their highest, specifically 0.90 and 1.00, respectively. (Figure 5e). The confusion matrix for the RF model and four representative trees with significant feature contributions are shown in Figures S22 and S23, Supporting Information. Therefore, there is an opportunity to increase the accuracy of ML decisions if the biomarker combinations are correctly selected. Figure 5e,f demonstrates that increasing the number of biomarkers improves the prediction accuracy of ML models. According to the results, the RF model consistently outperforms the NN model, regardless of whether single or multiple biomarkers are used, achieving higher prediction accuracy for sensing signals. Consequently, the RF model was chosen to analyze the original sensing data of clinical samples for more accurate pancreatic cancer screening. Although the RF model demonstrated satisfactory classification performance on the test set, we conducted tenfold cross‐validation to further assess its risk of overfitting and performed a hyperparameter grid search to identify the optimal parameters. The optimized model achieved a robust average accuracy of 0.955 ± 0.09 in the tenfold cross‐validation, indicating strong stability and good generalization ability across different data partitions. We have also analyzed the biomarkers (carbohydrate antigen 19‐9 (CA19‐9), carbohydrate antigen 242 (CA242), and carcinoembryonic antigen (CEA)) detection results provided clinically and compared them with the results obtained in this study (Table S7, Supporting Information). The results obtained through chemiluminescence immunoassay (CLIA) indicate that, compared with these traditional clinical biomarkers, the detection outcomes based on the three biomarkers (EGFR, GPC1, and MIF) demonstrate significant advantages. The Radar Chart provides a comparative analysis of various detection methods or technologies. It focuses on several key metrics including the limit of detection (LOD), total analysis duration, diagnostic accuracy/sensitivity (true positive rate)/specificity (true negative rate) and whether the sample needs pretreatment. Detailed information regarding these metrics is presented in Table S8, Supporting Information. As shown in Figure 5g, the key performance indicators and features of various detection methodologies can be visualized from the Radar Chart. Notably, the microsensor developed in this work stands out for its rapid response time, exceptional diagnostic accuracy, and simple process. These attributes render it a more convenient approach compared to the other methods listed.^[^ ^29^, ^30^, ^31^, ^32^ ^]^

The development of sensitive, specific, and user‐friendly diagnostic tools for cancer biomarker detection is critical for improving early diagnosis, guiding treatment decisions, and monitoring therapeutic responses. Some traditional methods often have limitations in terms of sensitivity and specificity, particularly when dealing with complex clinical samples like blood. In this study, integrating a MAIP detection platform with ML algorithms significantly enhanced the detection sensitivity and specificity. This is demonstrated by the fact that the developed approach can distinguish pancreatic cancer patients from acute pancreatitis patients and healthy controls with 100% accuracy. Moreover, the MAIP platform offers a streamlined workflow, reducing the need for extensive sample preprocessing or specialized equipment. Additionally, the integration of ML algorithms into the diagnostic process enhances the platform's interpretability and clinical utility. The modular design of the MAIP platform suggests its adaptability to other cancers and chronic diseases. By substituting disease‐specific antibodies, the same technology could be applied to detect biomarkers for conditions such as breast cancer, colorectal cancer, or neurodegenerative diseases, broadening its clinical impact.

## 3 Conclusion

In conclusion, we have successfully developed a MAIP‐based sensing platform that enables highly sensitive and specific detection of cancer biomarkers in human serum samples. The platform's exceptional performance is attributed to the strategic design of its sensory materials. By leveraging the exposed metal ion sites within the 2D Zn‐TCPP framework, the developed anti‐fouling sensing interface substantially enhances the detection capabilities of immunosensors. Through systematic optimization of the immobilization of antibody molecules and the anti‐fouling strategy of the sensing interface, the sensing platform demonstrates the capability to detect trace amounts of cancer markers, such as MIF, EGFR, and GPC1, within intricate biological media. Validation through clinical serum samples testing and ML analysis demonstrates the platform's potential for high diagnostic accuracy in cancer identification. The ML analysis revealed that the combination of GPC1 and EGFR, with low correlation, significantly improved the prediction accuracy for pancreatic cancer screening. Notably, the RF model consistently outperformed the NN model, making it the preferred choice for clinical sample analysis. Furthermore, increasing the number of biomarkers enhanced average prediction accuracy, reaching 100% when using a combination of three biomarkers. The versatility of this platform, combined with its ability to perform parallel detection of multiple tumor markers, can facilitate early and accurate diagnoses, enabling more effective treatment strategies tailored to individual patients.

## 4 Experimental Section

### Synthesis of 2D Zn‐TCPP/GO

2D Zn‐TCPP/GO composite was prepared using a seed‐assisted liquid phase growth strategy, wherein the preparation of 2D Zn‐TCPP referred to the literature.^[^ ^18^ ^]^ First, 10 mg of graphene oxide (GO) was dispersed into 25 mL of ethanol solution to form a homogeneous GO suspension by ultrasonication for 10 min. Then, the obtained dispersion liquid was deposited on the surface of a clean 1 × 1 cm^2^ silicon wafer with the assistance of a heating plate at 60 °C. Afterward, 250 µL of zinc acetate (Zn(CH_3_COO)_2_) ethanol solution (5 mM) was uniformly drop‐coated on the surface of the GO film, which was dried at room temperature and then calcined in a tube furnace at 350 °C for 20 min. After calcination, Zn(CH_3_COO)_2_ was converted to ZnO, and employed as the seeds for the oriented growth of 2D ultra‐thin Zn‐TCPP (TCPP = tetrakis(4‐carboxyphenyl)porphyrin). For the in situ growth of ultra‐thin Zn‐TCPP nanosheets, 22.5 mg of zinc nitrate hexahydrate (Zn(NO_3_)_2_·6H_2_O), 4.0 mg of pyrazine, and 100 mg of PVP (MW 40 000) were dissolved in a mixture of 60 mL of DMF and ethanol (3:1 (v/v)), which was recorded as solution A. Another solution B was prepared by dissolving 20 mg of TCPP into 20 mL of DMF/ethanol mixture (3:1 (v/v)). Solution B was added into solution A drop by drop under stirring. The obtained mixed solution was ultrasonicated for 10 min, and then sealed in a 100 mL reactor. The above‐mentioned silicon wafer was placed face down in the reactor and kept reaction at 80 °C for 16 h. After the reaction was finished, the silicon wafer was taken out and rinsed with ethanol for several times. The 2D Zn‐TCPP/GO composite was obtained after drying in a vacuum oven overnight at 60 °C. Besides, a bulk Zn‐TCPP sample was also synthesized, and served as a control.^[^ ^33^ ^]^ For0020the preparation, 44.5 mg of Zn(NO_3_)_2_·6H_2_O, 39.5 mg of TCPP, and 8 mg of pyrazine were added into 10 mL of DMF/ethanol solution (3:1 (v/v)), and stirred until fully dissolved. The resultant solution was transferred to a reactor and reacted at 80 °C for 24 h. The obtained purple precipitate was collected and washed with ethanol for three times, and then centrifuged at 11,000 rpm for 10 min to collect the sample.

### Material Characterization

The morphology of 2D Zn‐TCPP/GO sample was characterized by using a field‐emission scanning electron microscope (FE‐SEM, Regulus‐8230) from Hitachi, Japan. The ultra‐thin nature of Zn‐TCPP sample was confirmed by transmission electron microscope (TEM, JEM‐2100F) and atomic force microscopy (AFM, MultiMode8, Veeco). Elemental analysis was obtained by EDS which was equipped on SEM system. The formation of MOF structure was revealed by Empyrean Alpha 1 X‐ray diffractometer (PANalytical) and Nicolet iS50 FT‐IR spectrometer (Thermo Fisher).

### X‐ray Absorption Fine Structure Characterization and Analysis

The XAFS spectra of Zn K‐edge of samples were measured at the BL14W1 beamline by using Si (111) crystal monochromators in Shanghai Synchrotron Radiation Facility, China. Prior to analysis at the beamline, the samples were pressed into thin sheets with the diameter of 1 cm and sealed with Kapton tape film. The XAFS spectra were recorded at room temperature using a 4‐channel silicon drift detector (SDD, Bruker 5040). EXAFS were recorded in transmission mode, and negligible changes in the line shape and peak position of the Zn K‐edge XANES spectra were observed between two scans of a specific sample.

Data approximation, data analysis and EXAFS fitting were performed by Athena and Artemis software.^[^ ^34^ ^]^ The energy calibration of the samples was carried out by using a standard zinc foil, which was also used as a reference for the measurements. The extracted EXAFS data was weighted by k^3^, and converted to R‐space by Fourier transforms (FT) to obtain the global amplitude EXAFS (CN, R, σ^2^ and ΔE_0_). The value of the amplitude reduction factor S_0_^2^ was determined by fitting the reference zinc foil, and set in EXAFS analysis to determine the CNs. The Debye‐Waller factors and ΔRs were obtained by constraining the Zn‐O/N on the basis of the Guessing‐Parameter, and the Wavelet Transformation (WT) was performed using the software developed by Funke and Chukalina, with the Morlet Wavelet κ = 10, σ = 1.^[^ ^35^, ^36^ ^]^

### Density Functional Theory Calculations

DFT calculations were performed using the Vienna Ab initio Simulation Package (VASP). The projector augmented‐wave (PAW) method implemented in the VASP code, and was utilized to describe the ion‐electron interaction.^[^ ^37^, ^38^ ^]^ The generalized gradient approximation (GGA) parameterized by Perdew, Burke, and Ernzerhof (PBE) was employed to describe the exchange‐correlation potential.^[^ ^39^ ^]^ The weak van der Waals interactions were calculated using the DFT‐D3 dispersion correction method.^[^ ^40^, ^41^ ^]^ The strongly correlated effect of d orbits of Zn was modified by DFT+U with 7.5 eV of U value. An energy cutoff of 450 eV and 1 × 1 × 1 at the k‐point in the Brillouin zone were used for structure optimization. The convergence thresholds for energy and maximum stress were 10^−5^ eV and 0.02 eV Å^−1^, respectively. The density of states was calculated by tetrahedral method with Blöchl correction, using the 2*2*2 of k‐points.

### Fabrication of Electrochemical Sensing Chip

On the basis of the standard three‐electrode system (reference electrode, working electrode and counter electrode), a five‐electrode system with three working electrodes was designed. The silver/carbon ink for the working and counter electrodes, the Ag/AgCl ink for the reference electrode, and the silver ink for the conductive wires were screen‐printed onto a PET flexible substrate to obtain electrochemical sensing electrodes that can perform the three‐channel parallel detection. The prepared 2D Zn‐TCPP/GO electrode material (4 mg) was homogeneously dispersed into a mixture of 100 µL ethanol and 50 µL Nafion 117, and then locally deposited on the working electrodes. A resin template was printed using a high‐resolution DLP/SLA 3D printer (nanoArch S140) and the PDMS microchannels were fabricated with the assistance of the template. The PDMS channels and PET‐based electrochemical sensing electrodes were well‐sealed by air plasma treatment using a PDC‐002‐HP Plasma Cleaner (HARRICK PLASMA) under 45 W of treating power in vacuum for 90 s to produce the electrochemical sensing chip.

### Construction and Characterization of Antifouling Surfaces

After fabricating the electrochemical sensing chip, the construction of the antifouling sensing interface proceeded through the following steps. The lyophilized powder peptide was first dissolved in phosphate buffer saline (PBS, pH = 7.4) solution to prepare a peptide PBS solution with a concentration of 0.25 mg mL^−1^. Then, 25 µL of peptide PBS solution was injected into the channel through each of the three sample inlets and incubated at room temperature for 24 h. After incubation, the channels were washed with PBS solution to remove the residual peptide molecules.

Next, this work used three proteins with different charges (positive, negative, and neutral) as well as real serum to evaluate the anti‐fouling performance of the peptides. During the experiment, 25 µL of the testing protein solution was injected into the channels and allowed to incubate at 37 °C for 1 h. After incubation, the residual protein solution was washed away, and subsequently, 100 µL of PBS solution was injected into the microchannels for electrochemical measurements. Finally, the antifouling performance was evaluated through DPV testing. The degree of peak current attenuation was used as an indicator to determine the antifouling capability of developed sensing platform.

In addition to electrochemical characterization, X‐ray photoelectron spectroscopy (XPS, Thermo Scientific K‐Alpha) was employed to characterize the 2D Zn‐TCPP/GO‐peptide surface. Furthermore, an XG‐CAMC3 dynamic contact angle meter was used to measure the static contact angle of electrode before and after peptide modification. Additionally, Zeta potential of the peptide solution was measured using a Malvern Zetasizer Nano ZS90 instrument.

### Electrochemical Immunosensing Test

A volume of 100 µL of Protein A solution (10 µg mL^−1^) was injected into the microchannels and allowed to incubate at 4 °C overnight. The redundant protein A solution was removed and the channels were washed with PBS+0.05% Tween 20 for three times. After incubating protein A, 25 µL of Anti‐GPC1 antibody/PBS, Anti‐EGFR antibody/PBS and Anti‐MIF antibody/PBS with the concentration of 10 µg mL^−1^ was separately injected into three testing channels connected to the working electrodes and incubate at 37 °C for 1 h. The washing buffer (PBS+0.05% Tween 20) was used to remove the un‐bonded antibodies. Subsequently, 100 µL of anti‐fouling peptide solution (0.25 mg mL^−1^) was injected and allowed to incubate at room temperature for 24 h to block non‐specific protein adsorption sites. Afterward, the testing channels were washed three times with washing buffer. Following this procedure, 100 µL of the testing solution was injected and incubated for 30 min at 37 °C. Subsequently, the solution was aspirated, and the testing channels are washed with washing buffer to remove any residual solution. After that, 100 µL of PBS buffer was injected into the microchannels for testing.

Electrochemical impedance measurements were performed to characterize the processes involved in the immunoreaction at amplitude of 10 mV in the region of ≈10^−2^–10^5^ Hz. DPV was used to measure the electrochemical response of the developed MAIP‐based platform toward protein markers. All electrochemical tests were conducted on an electrochemical workstation (BioLogic, VSP‐300).

### Clinical Samples Testing

Human serum samples were collected from 32 pancreatic cancer patients, 12 acute pancreatitis patients and 20 healthy donors at Shanghai Tongji Hospital, Shanghai. All participants provided informed consent for the use of their specimens in this research study. The clinical samples utilized in this study were processed following a standardized protocol, which involved centrifuging the blood to obtain serum, without removing exosomes. The relative centrifugal force (RCF) applied during centrifugation was 1500 × g, with a duration of 10 min, conducted at room temperature.

First, serum samples were diluted tenfold using PBS to prepare them for subsequent analysis. The electrochemical immuno‐sensing procedure of serum samples followed the description in electrochemical immunosensing test section. After incubating with the anti‐fouling peptide, 100 µL of diluted clinical serum samples were injected into microchannels and incubated at 37 °C for 30 min. Subsequently, the chip was washed three times with washing buffer to remove unbound protein molecules. Following the washes, 100 µL of PBS solution was added to the chip, and DPV were employed for detection.

### Machine Learning Analysis

This research employed RF and NN algorithms from the Scikit‐learn library, alongside multiple open‐source libraries including Pandas, Matplotlib, and NumPy. The RF algorithm consisted of 100 random decision trees, used for binary classification of electrochemical signals from clinical samples. The NN model comprised an input layer (with three neurons), two hidden layers (with 200 and 50 neurons, respectively), and an output layer. Both models performed analyses on raw data (balanced data set) under the framework of supervised learning. Initially, 70% of the dataset was randomly allocated for training using stratified sampling, and the remaining 30% is used to validate the prediction performance of the algorithms. The current signals of three biomarkers were input into the RF and NN models. The ML models then evaluated the signal combinations and ultimately output prediction results.

## Conflict of Interest

The authors declare no conflict of interest.

## Supporting information



Supporting Information

## Data Availability

The data that support the findings of this study are available from the corresponding author upon reasonable request.
