# Supplementary material for: An MOF‐Enhanced Anti‐Fouling Immunoprobe Platform for Efficient Direct Screening of Pancreatic Cancer
Source: Adv Sci (Weinh). 2025 Jul 1;12(36):e03619. doi: 10.1002/advs.202503619 (PMC12463068; doi:10.1002/advs.202503619)
Supplement: Supplementary file 1 — Supporting Information [file ADVS-12-e03619-s001.docx]

Supporting Information

An MOF-enhanced Anti-fouling Immunoprobe Platform for Efficient Direct Screening of Pancreatic Cancer

Wenjuan Yang^1,†^, Shujuan Mao^1,†^, Hao Zhu^1^, Handong Yao^1^, Wei Jin^1^, Lingyan Feng^1^, Xinghua Gao^1^, Xuefeng Wang^2^, Wei Chen^3^, Min Tu^2^*, Yuan Zhang^1^*

W. Yang, S. Mao, H. Zhu, H. Yao, W. Jin, L. Feng, X. Gao, Y. Zhang

Materials Genome Institute, Shanghai Engineering Research Center for Integrated Circuits and Advanced Display Materials

Shanghai University

Shanghai 200444, China

E-mail: zhangyuan@shu.edu.cn

X. Wang, M. Tu

State Key Laboratory of Transducer Technology

Shanghai Institute of Microsystem and Information Technology, Chinese Academy of Sciences

Shanghai 200050, China

E-mail: min.tu@mail.sim.ac.cn

W. Chen

Department of Emergency

Tongji Hospital, Tongji University School of Medicine

Shanghai 200065, China

**Table of Contents**

[**Supplementary Figures** 2](#_Toc190272072)

[**Supplementary Tables** 16](#_Toc190272073)

[**Supplementary References** 23](#_Toc190272074)

**Supplementary Figures**

**
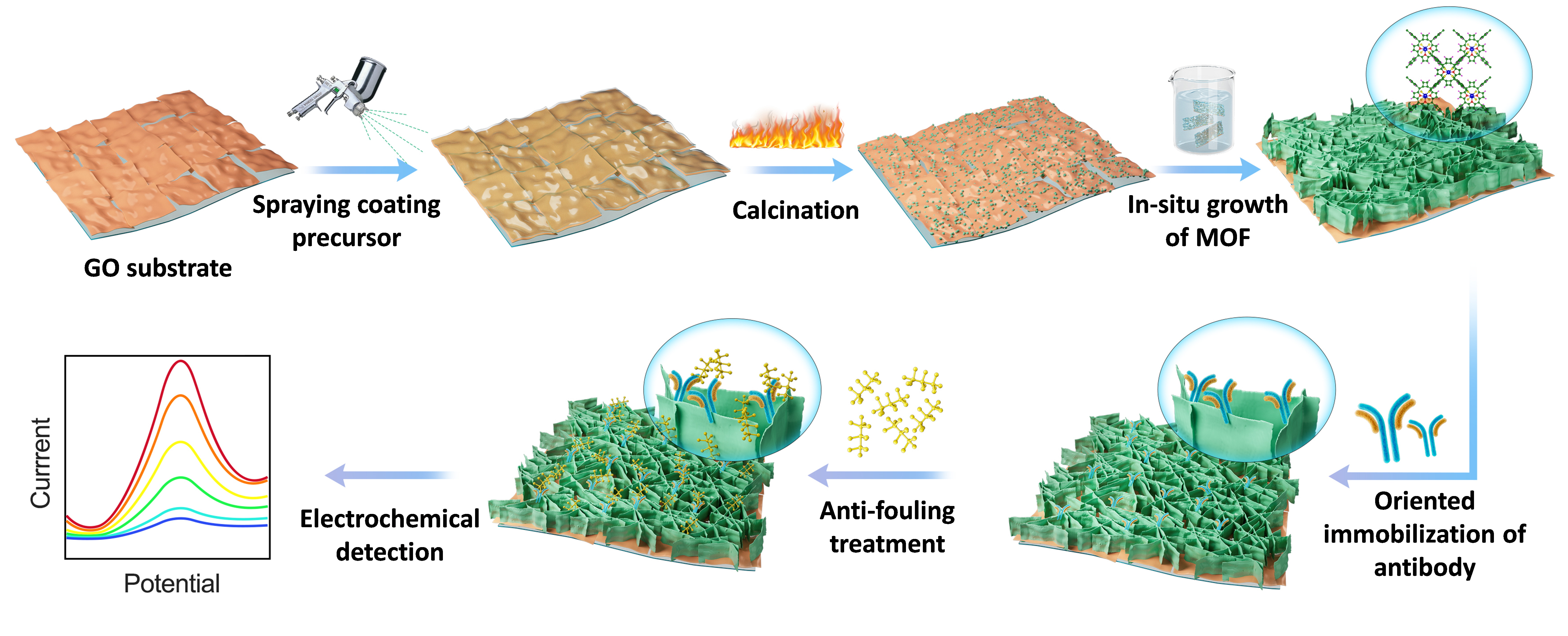
**

**Figure S1.** Schematic illustrating the process of creating a self-standing Zn-TCPP nanostructure for anti-fouling treatment and detecting protein biomarkers.

Zn-TCPP nanosheets were in situ grown on the surface of graphene oxide (GO) through a seed-assisted liquid phase growth strategy. Firstly, GO suspension was deposited on a clean silicon wafer. Then, zinc acetate (Zn(CH_3_COO)_2_) ethanol solution was evenly sprayed onto GO film and converted into ZnO seeds after calcination. Under liquid phase growth conditions, ultrathin Zn-TCPP nanosheets were in situ grown on the GO surface to form a 2D Zn-TCPP/GO composite nanostructure. Herein, we mainly utilize the abundant active sites in the 2D Zn-TCPP/GO composite material to achieve the antibody immobilization and anti-fouling treatment. Histidine (His)-tagged protein A has a high affinity with transition metal ions, allowing for stable attachment to Zn-TCPP nanosheets, and then the specific binding between Protein A and the Fc region of antibody could be used to for the oriented immobilization of recognition antibody. The residual metal ions are used for the attachment of anti-fouling peptide molecules by binding thiol group of the cysteine residues in peptide sequencing. The fully oriented-immobilization of recognition antibody provides the correct spatial configuration for specifically capturing target proteins, and anti-fouling surface constructed with peptide molecules reduces non-specific protein adsorption, ensuring detection sensitivity in complex serum matrix.


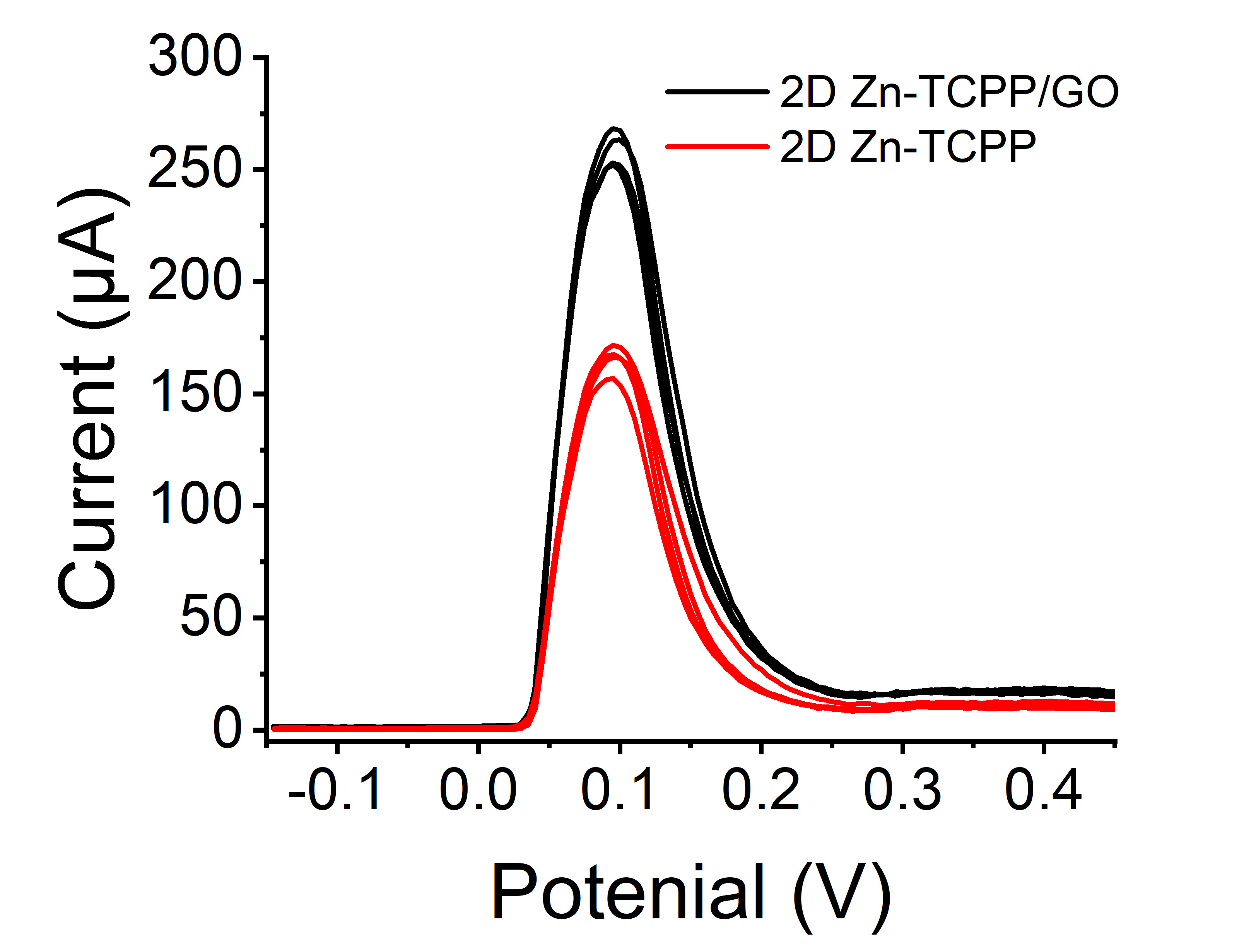


**Figure S2.** DPV responses of 2D Zn-TCPP/GO and 2D Zn-TCPP modified electrodes.


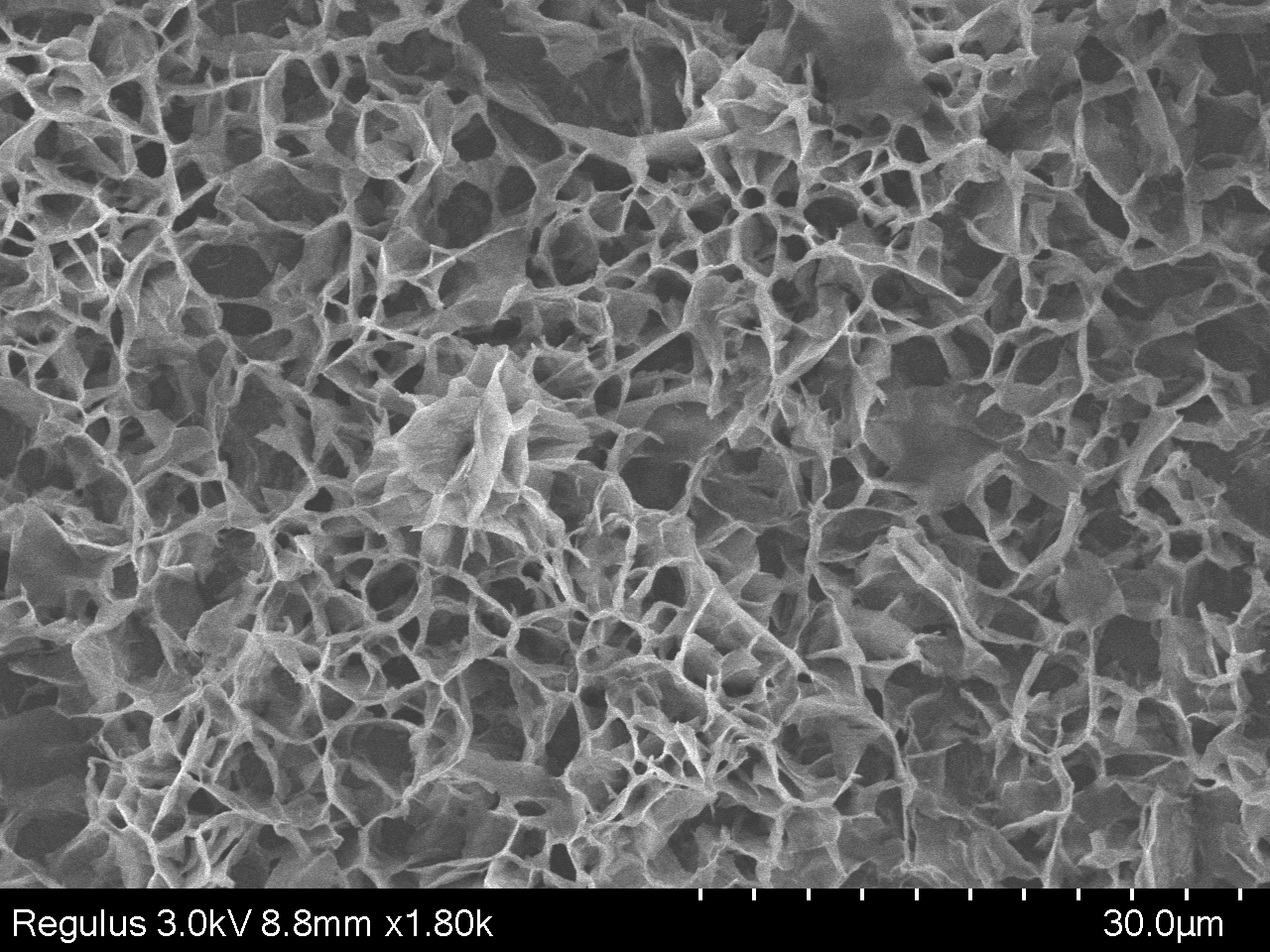


**Figure S3.** SEM image of 2D Zn-TCPP/GO nanocomposite after being immersed in PBS (pH=7.4) for 24h.


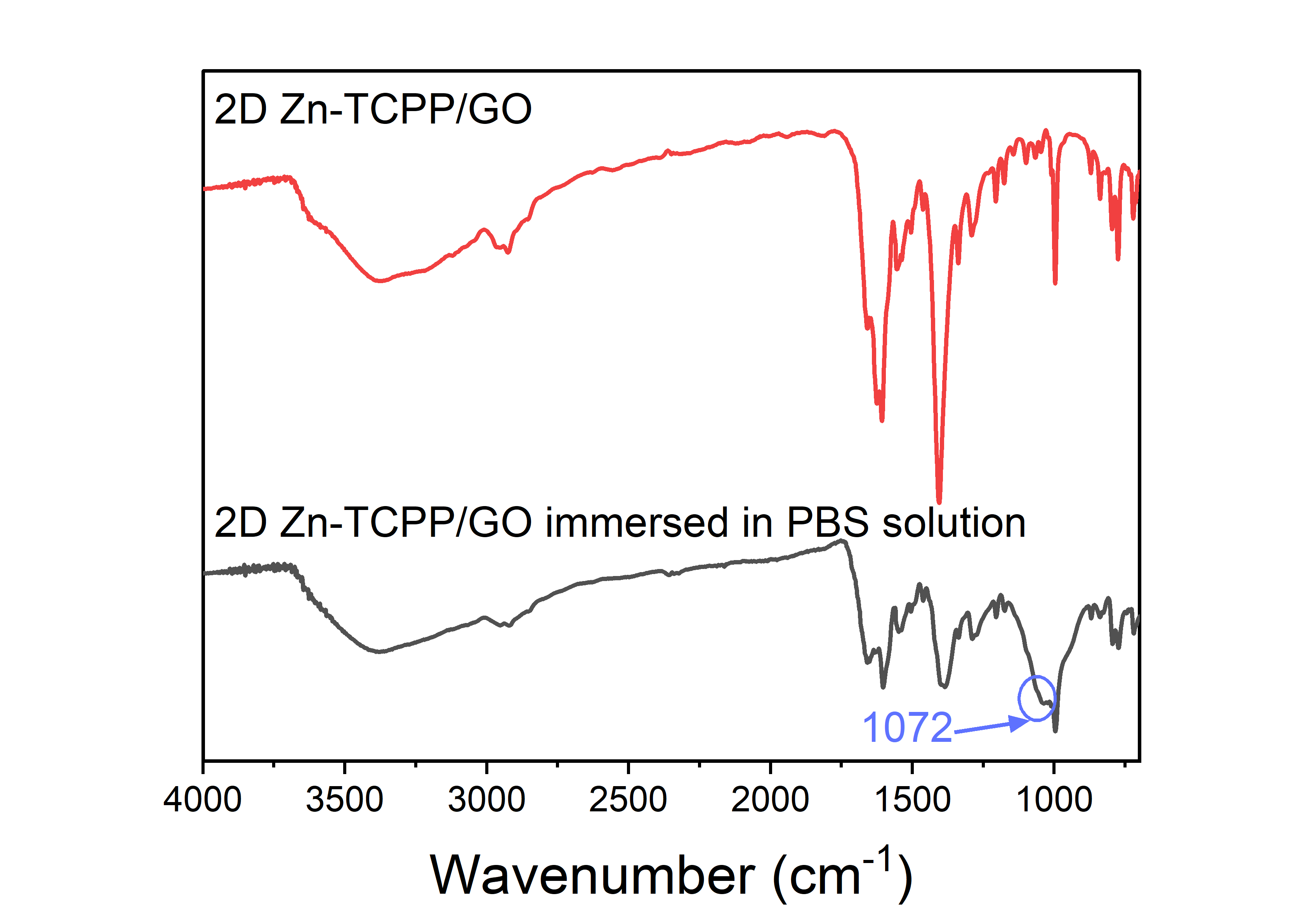


**Figure S4.** FT-IR spectrum of 2D Zn-TCPP/GO nanocomposite after being immersed in PBS solution (pH = 7.4) for 24h.


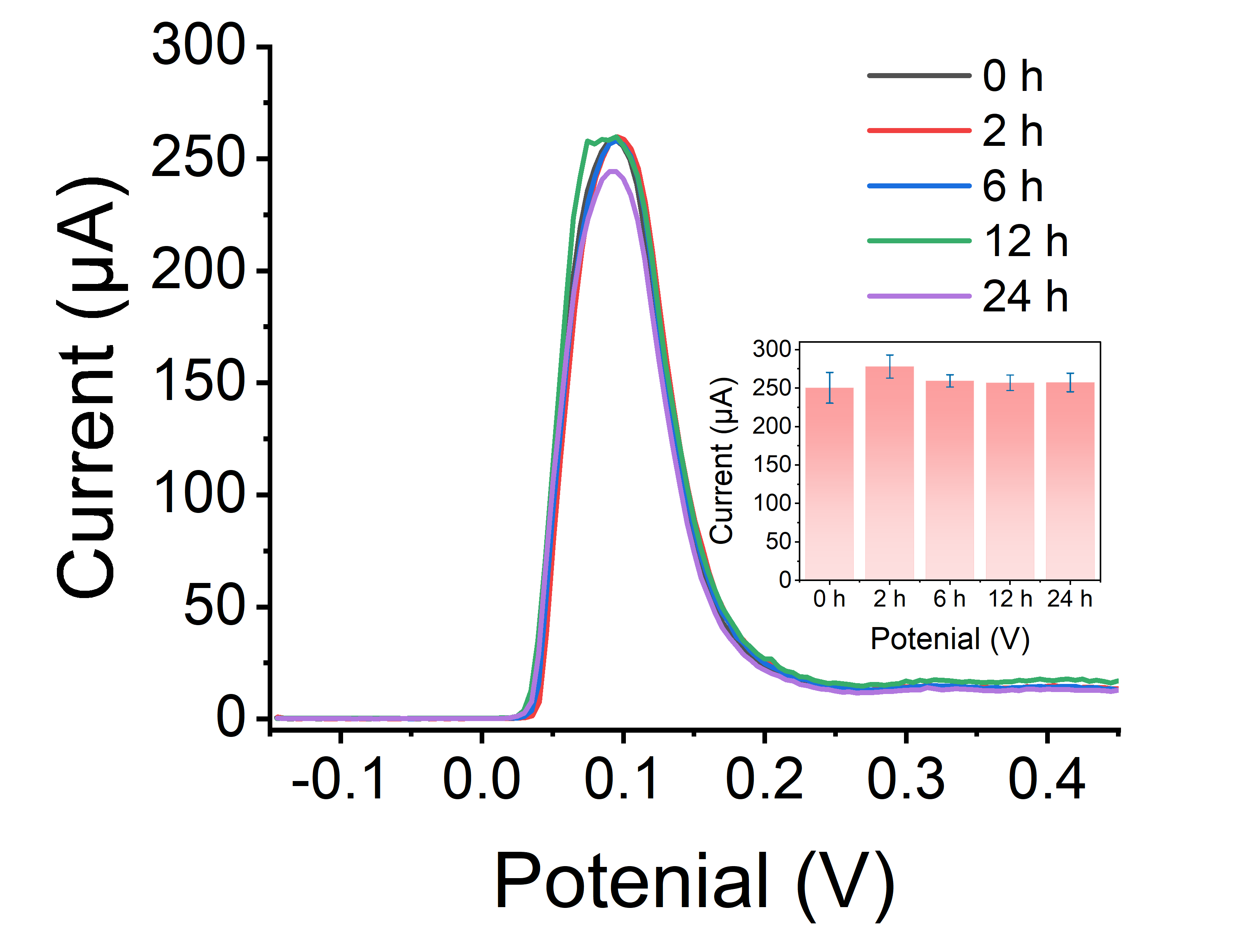


**Figure S5.** DPV results of 2D Zn-TCPP/GO nanocomposite at different immersion times (2h, 6h, 12h, and 24h) in PBS solution (pH = 7.4).





**Figure S6**. FT-IR spectra of Zn-TCPP/GO composite.

Fourier transform infrared spectroscopy (FT-IR) is used to confirm the formation of the 2D MOF structure. From the FT-IR spectrum of the TCPP ligand, the absorption peaks at around 3460 cm^-1^ and 3317 cm^-1^ are attributed to the O-H stretching vibration of the carboxyl group and the N-H stretching vibration on the porphyrin ring, respectively.^[1]^ These two characteristic peaks disappeared in the spectra of bulk Zn-TCPP, 2D Zn-TCPP and 2D Zn-TCPP/GO samples, indicating that the O and N atoms in the TCPP ligand coordinate with Zn^2+^ to form Zn-N/O coordination bonds. In addition, in the spectra of bulk Zn-TCPP, 2D Zn-TCPP and 2D Zn-TCPP/GO composites, a new absorption peak appeared at 1000 cm^-1^, while the N-H in-plane vibration peak at 965 cm^-1^ disappeared, as well as the C=O stretching vibration at 1688 cm^-1^ and porphyrin skeleton vibration peaks (1604, 1177, and 1100 cm^-1^) weakened, all indicating that the Zn–TCPP structure is a metalloporphyrin.^[2]^


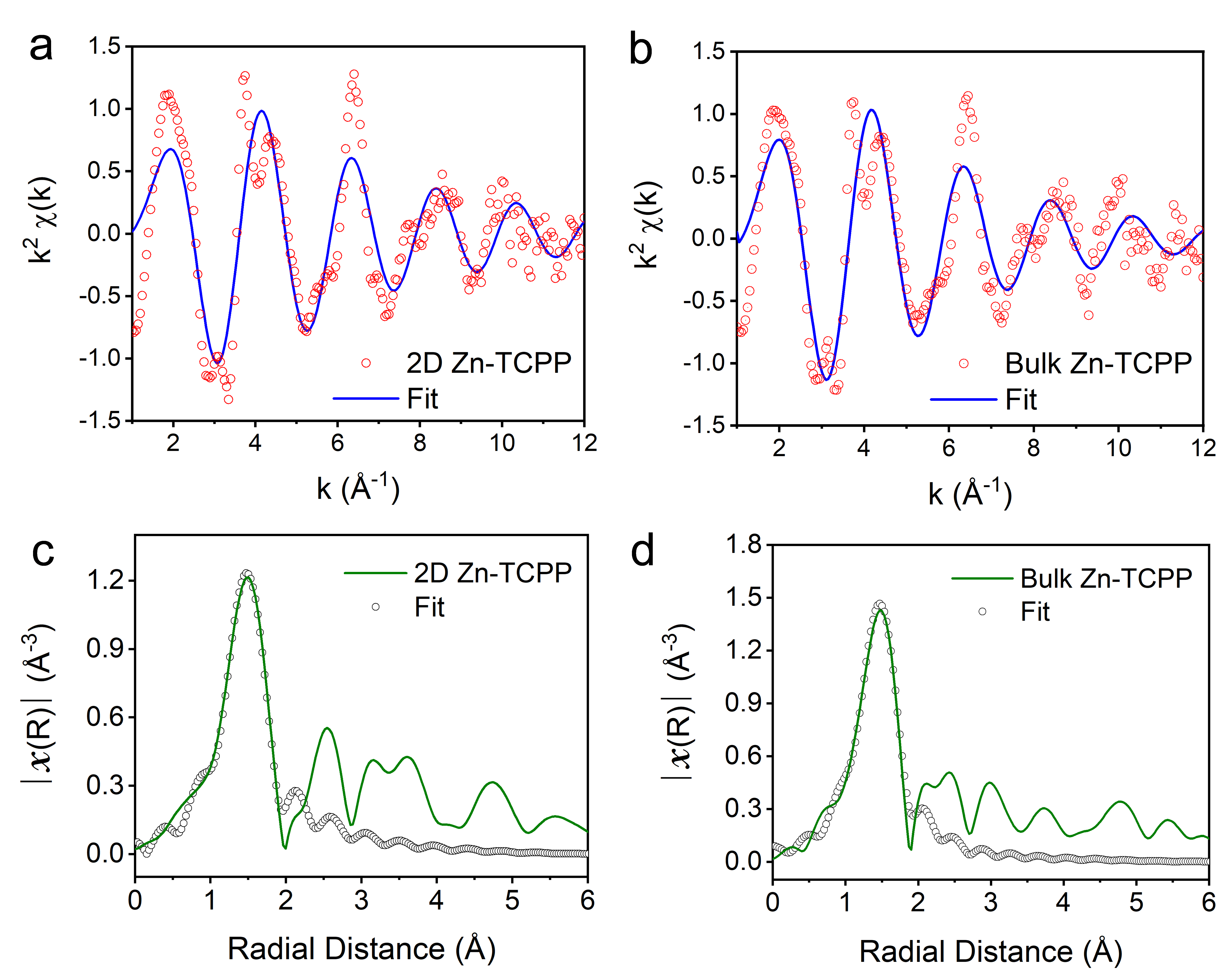


**Figure S7.** (a-b) Zn K-edge EXAFS (points) and fit (line) for 2D Zn-TCPP and bulk Zn-TCPP, shown in k^2^ weighted *k*-space. (c-d) Fitting results in R-space after the Fourier transform.





**Figure S8.** (a-b) The total DOS for 2D Zn-TCPP and bulk Zn-TCPP, and Zn, O, H, N and C are the partial density of states of each element. All the Fermi levels have been set at zero. The true value of the Fermi energy level is E_f_. (c) DPV current responses of 2D Zn-TCPP/GO and bulk Zn-TCPP/GO-based sensors.

**
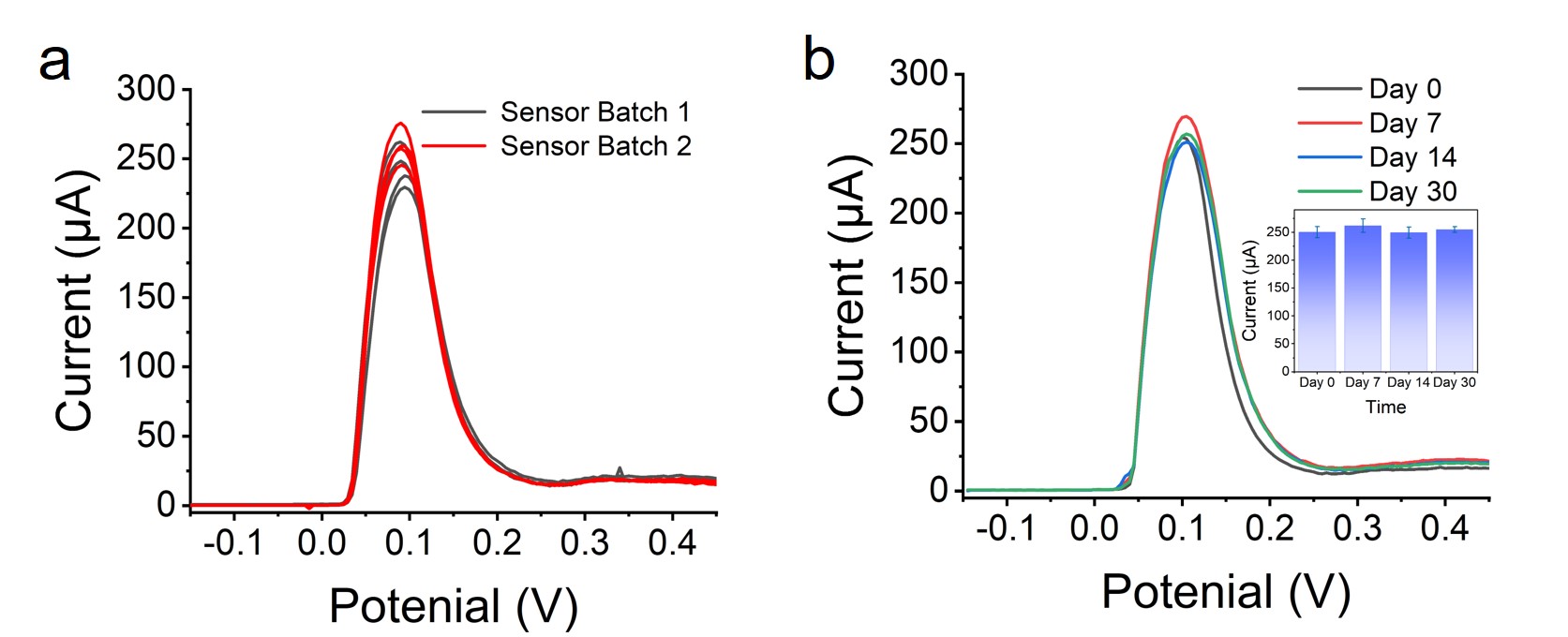
**

**Figure S9.** DPV responses for batch-to-batch reproducibility (a) and long-term storage stability (b) of 2D Zn-TCPP/GO modified electrodes.


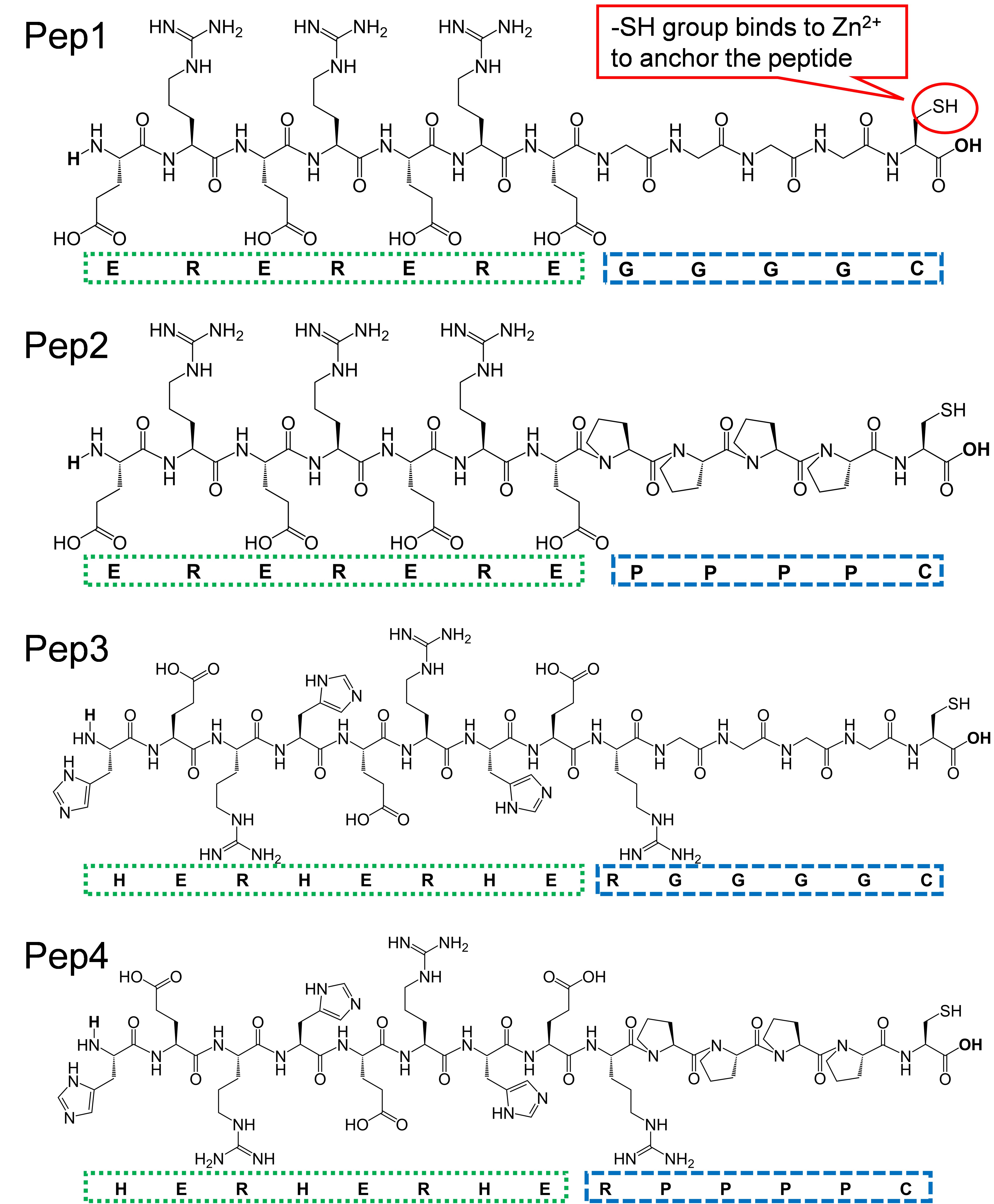


**Figure S10.** The molecular structures of four peptide molecules. The designed peptides are described as EREREREGGGGC (Peptide 1, Pep 1), EREREREPPPPC (Peptide 2, Pep 2), HERHERHERGGGGC (Peptide 3, Pep 3), HERHERHERPPPPC (Peptide 4, Pep 4), respectively. It includes the antifouling part (green dotted box) and the anchoring part (blue dotted box).


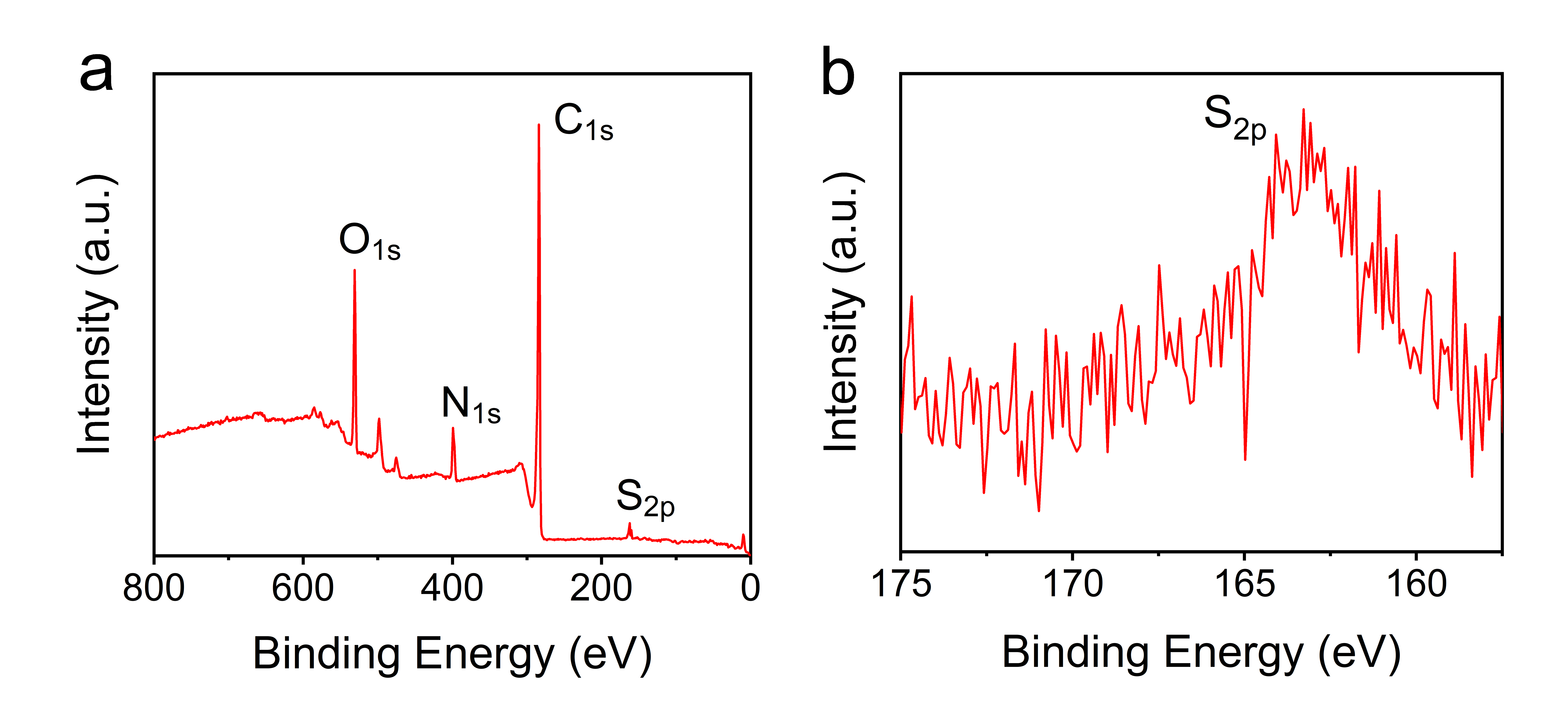


**Figure S11.** X-ray photoelectron spectroscopy (XPS) survey spectra of (a) peptide molecules modified 2D Zn-TCPP/GO and (b) corresponding XPS high-resolution spectra for S_2p_. The S_2p_ characteristic peak at about 162 eV is originated from sulfur (S) element of -SH group at the peptide’s C-terminus, which indicates that the peptide molecules are successfully coupling with the sensing interface indicating the feasibility of the proposed strategy.


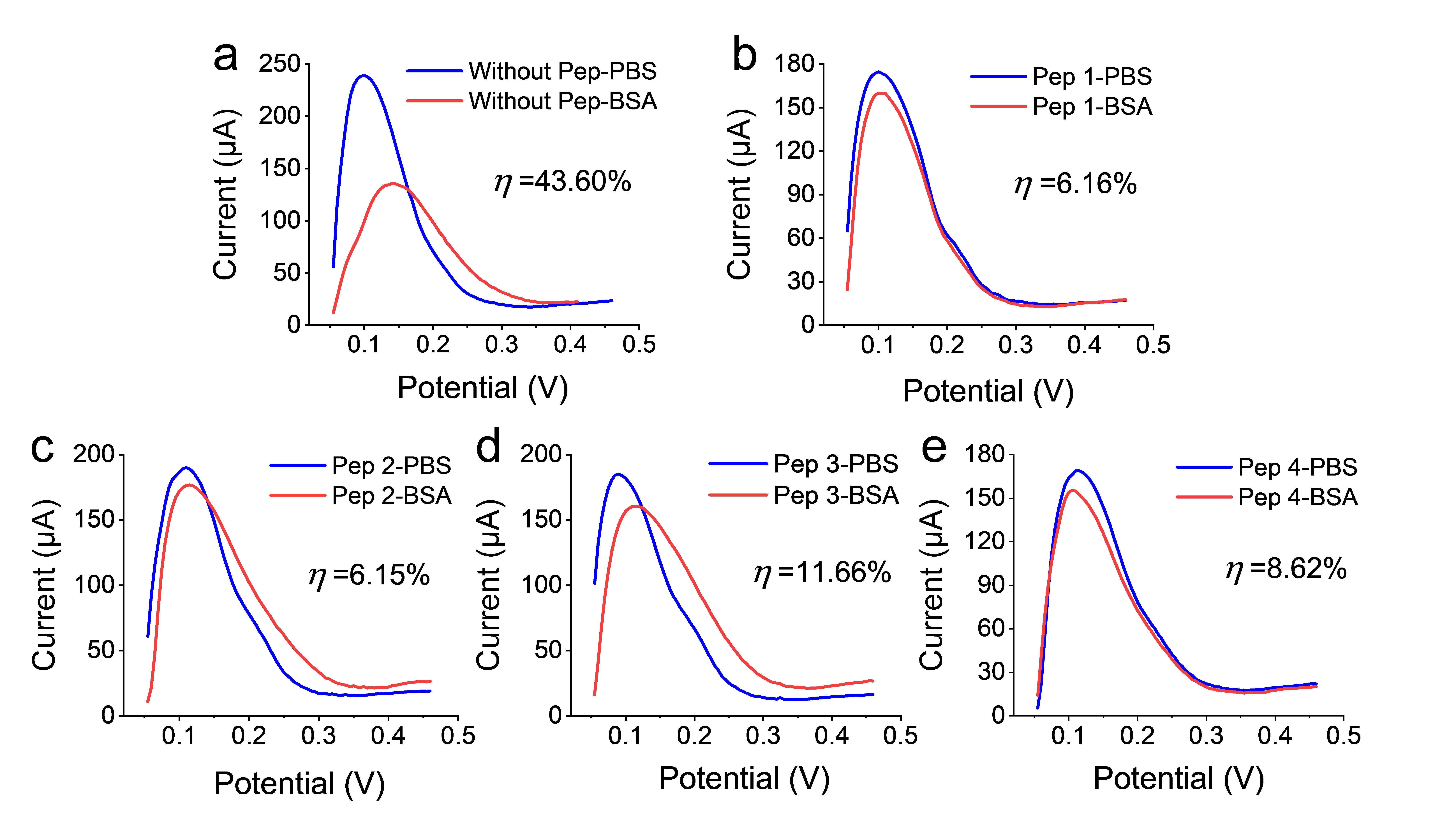


**Figure S12.** Differential pulse voltammetry (DPV) testing curves of electrodes modified with and without peptides in BSA protein environment. The attenuation percentage (η) of DPV currents of the electrochemical sensor chip in blank PBS solution and PBS-BSA solution (5 mg/mL) is used to evaluate the anti-fouling of different peptides, and $\eta=-\Delta I/I_{0}$, $\Delta I=I-I_{0}$, where I_0_ is the peak current tested in bland PBS solution, and I is the peak current tested in PBS-BSA solution.


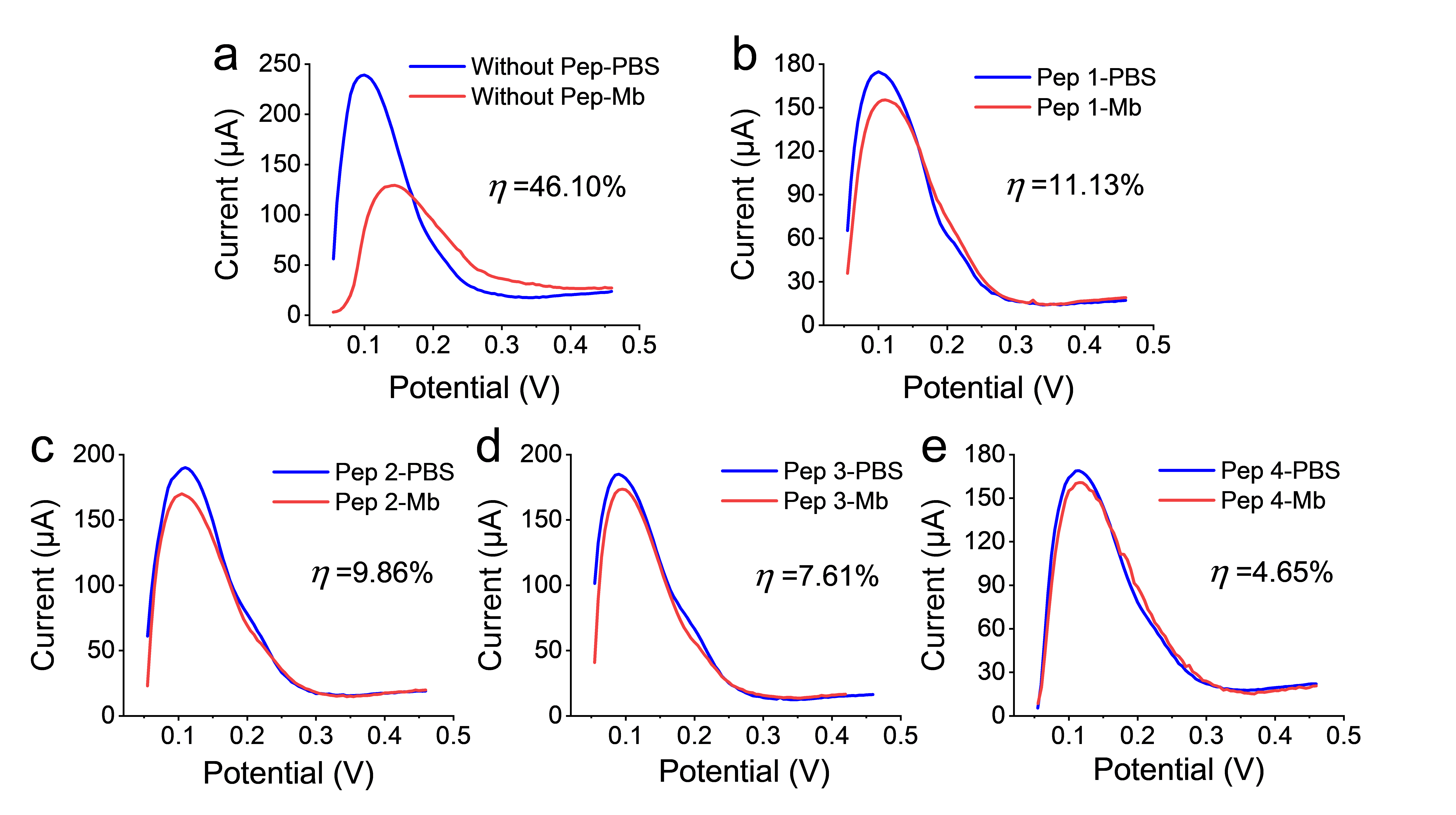


**Figure S13.** DPV testing curves of electrodes modified with and without peptides in Mb protein environment.


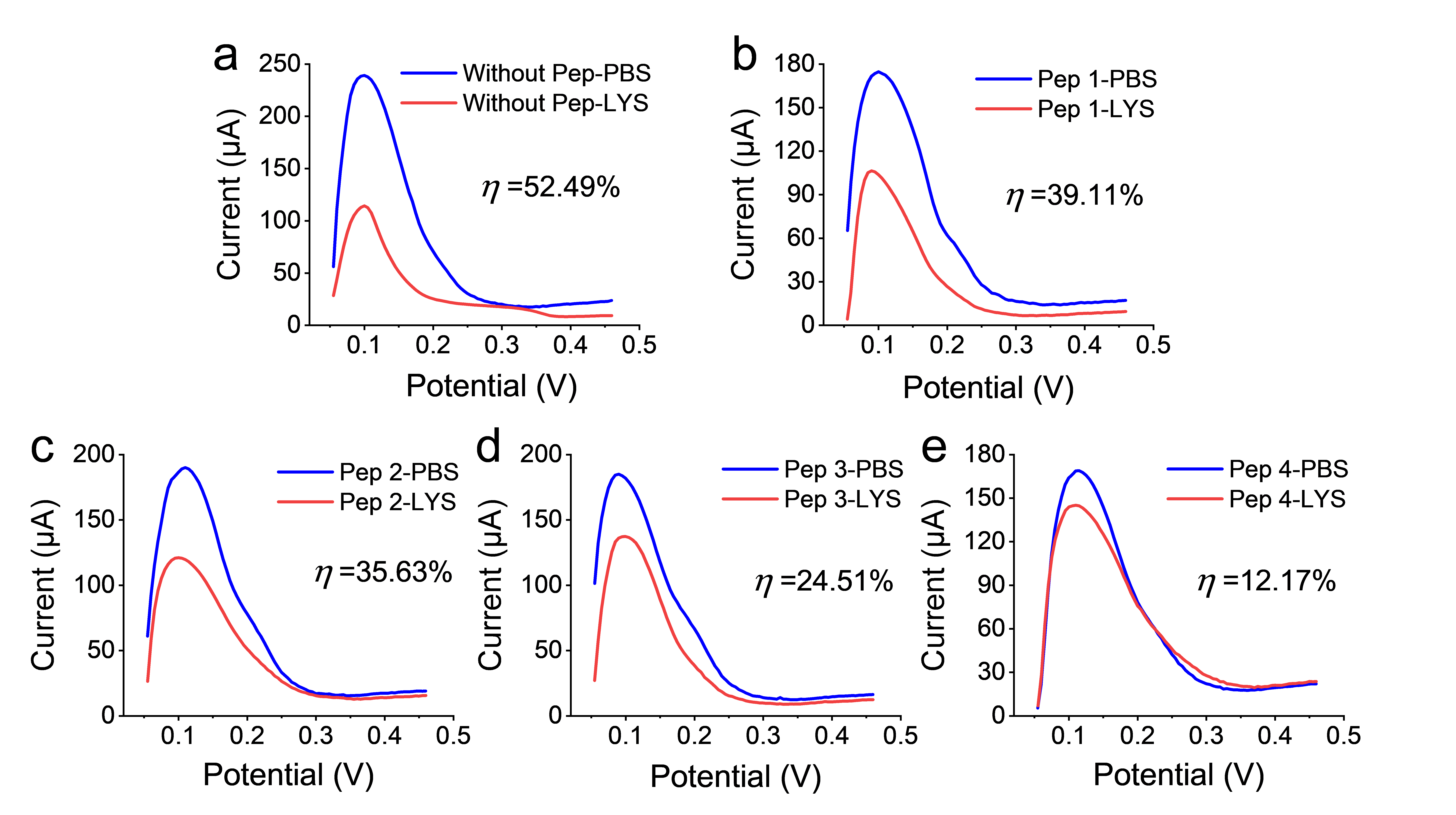


**Figure S14.** DPV testing curves of electrodes modified with and without peptides in LYS protein environment.


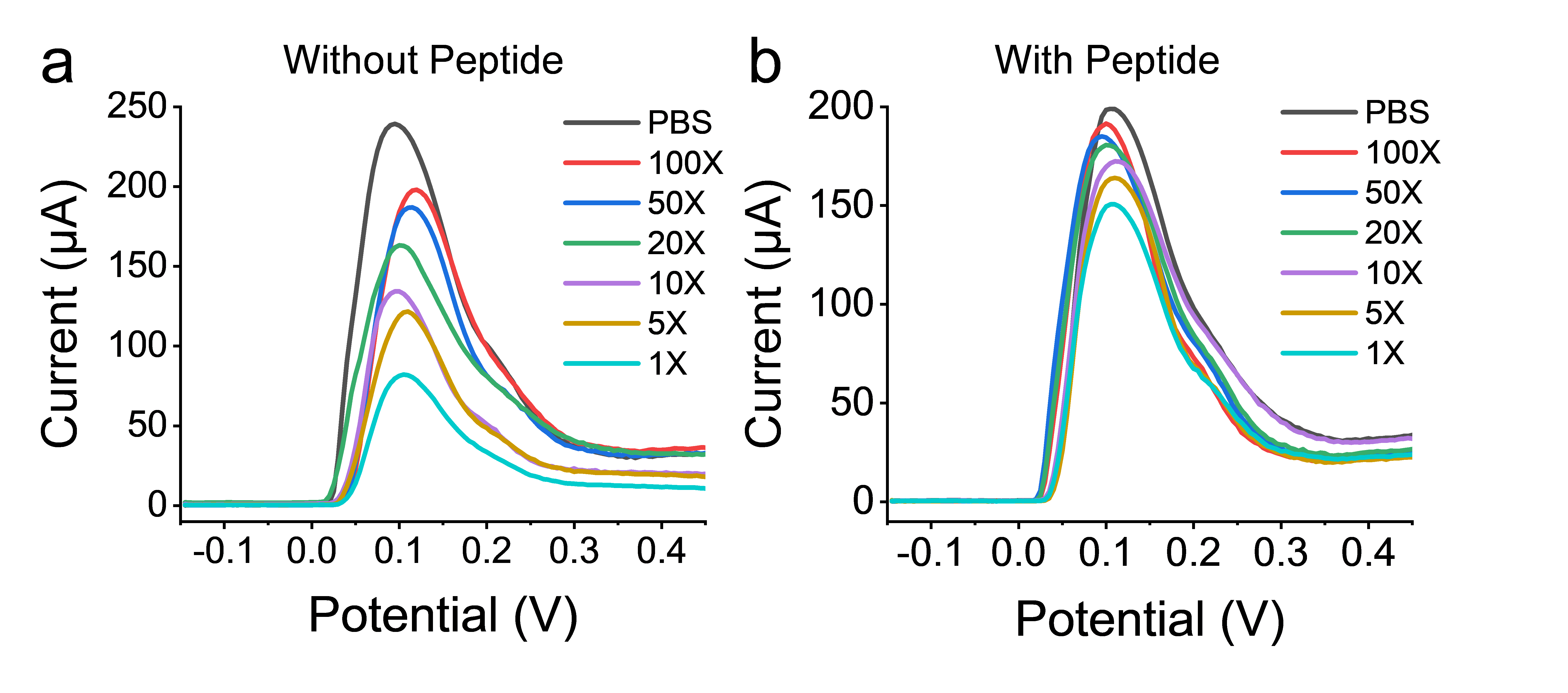


**Figure S15.** DPV testing curves of electrodes modified with pep 4 in real serum diluted with different multiples.


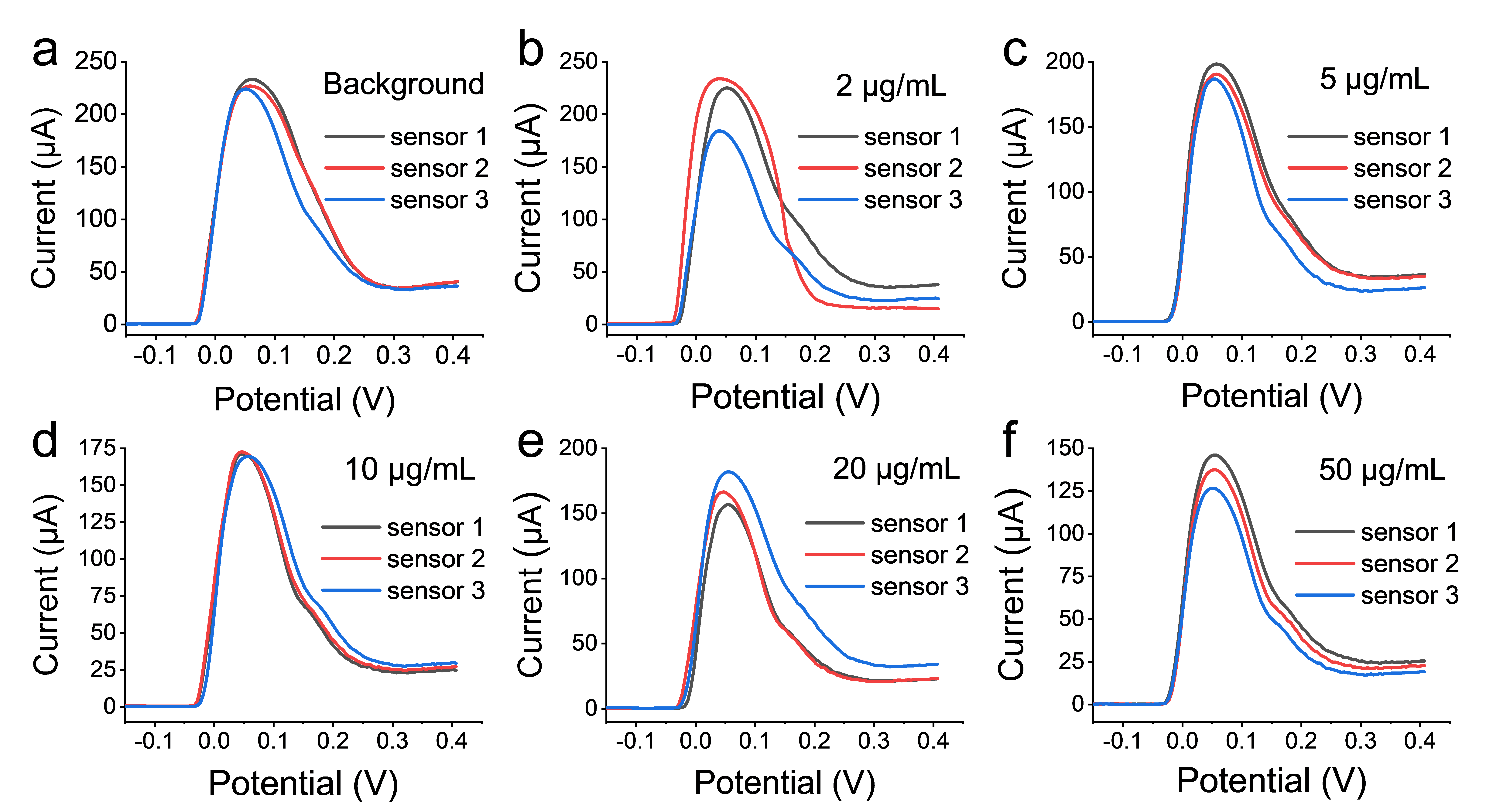


**Figure S16.** Evaluation of the optimal ratio between protein A and Goat Anti-Human IgG. The concentration of protein A was fixed at 10 μg/mL, and the concentrations of Goat Anti-Human IgG were 2 μg/mL (protein A: Goat Anti-Human IgG = 5:1), 5 μg/mL (protein A: Goat Anti-Human IgG = 2: 1), 10 μg/mL (protein A: Goat Anti-Human IgG =1:1), 20 μg/mL (protein A: Goat Anti-Human IgG =1:2) and 50 μg/mL (protein A: Goat Anti-Human IgG =1: 5), respectively. The DPV signal was recorded after incubating with 1 μg/mL of human IgG. According to the results, the optimal ratio of protein A to goat anti-human IgG was determined to be 1:1.


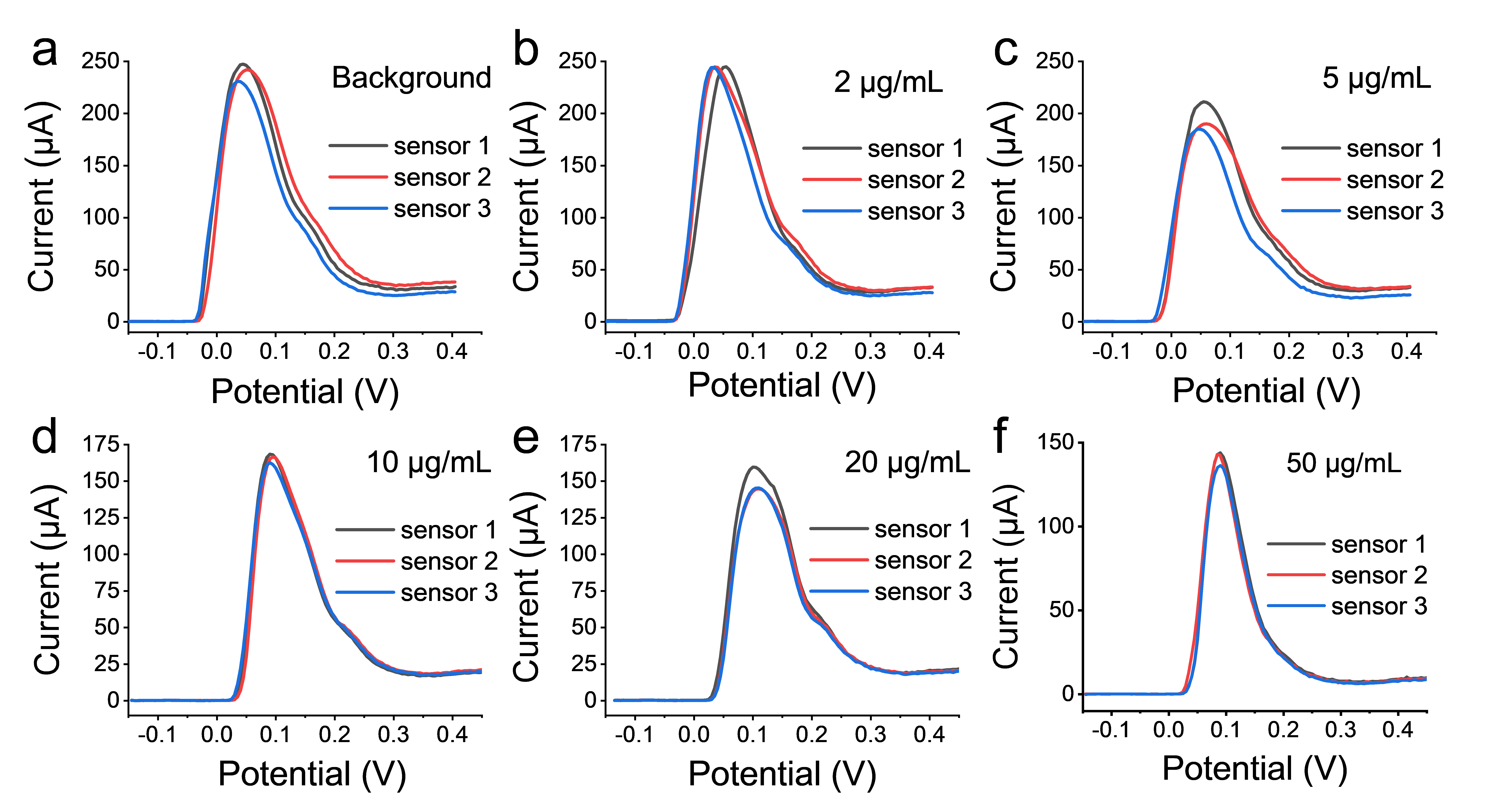


**Figure S17.** The optimization of goat anti-human IgG concentrations for immunoreaction. The concentrations of goat anti-human IgG (maintained at a fixed 1:1 ratio with protein A) were 2 μg/mL, 5 μg/mL, 10 μg/mL, 20 μg/mL and 50 μg/mL, respectively. The DPV signal was recorded after incubating with 1 μg/mL of human IgG. The results indicated that a more significant output signal was achieved when the concentration of goat anti-human IgG was 10 μg/mL.


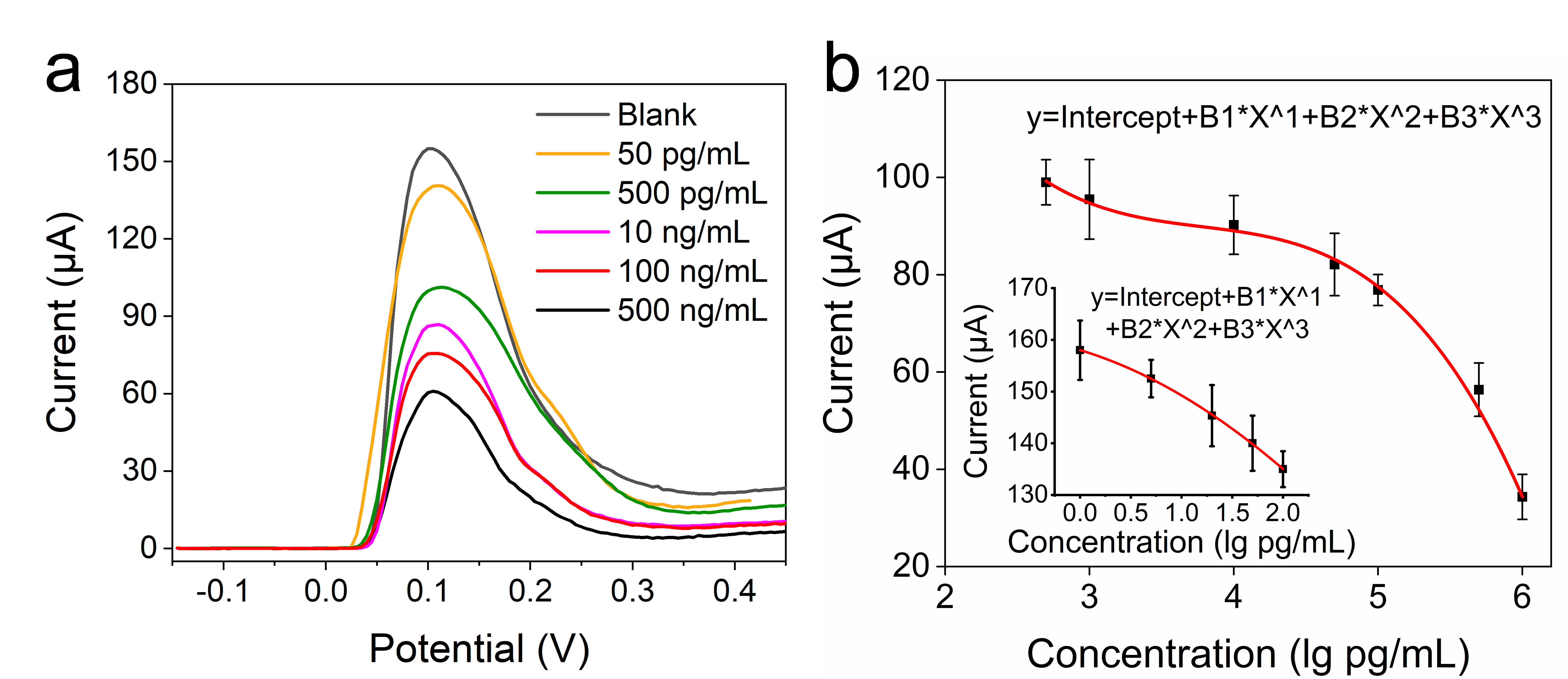


**Figure S18.** DPV measurements of the developed microsensor in the presence of human IgG at various concentrations as well as corresponding dose-dependent response results. The decrease in peak current with increasing human IgG concentration is due to the enhanced interfacial electronic resistance caused by the formation of immune complexes. The equation and corresponding parameters for the dose-dependent response of IgG are presented below.

|  | 5-100 pg/mL | 500 pg/mL-1000 ng/mL |
| --- | --- | --- |
| equation | y = Intercept + B1*x^1 + B2*x^2 + B3*x^3 | |
| Intercept | 158.0122 | 337.0180 |
| B1 | -5.8814 | -188.9303 |
| B2 | -2.9570 | 49.0387 |
| B3 | 0.0755 | -4.3262 |
| Adjusted R² | 0.9995 | 0.9974 |


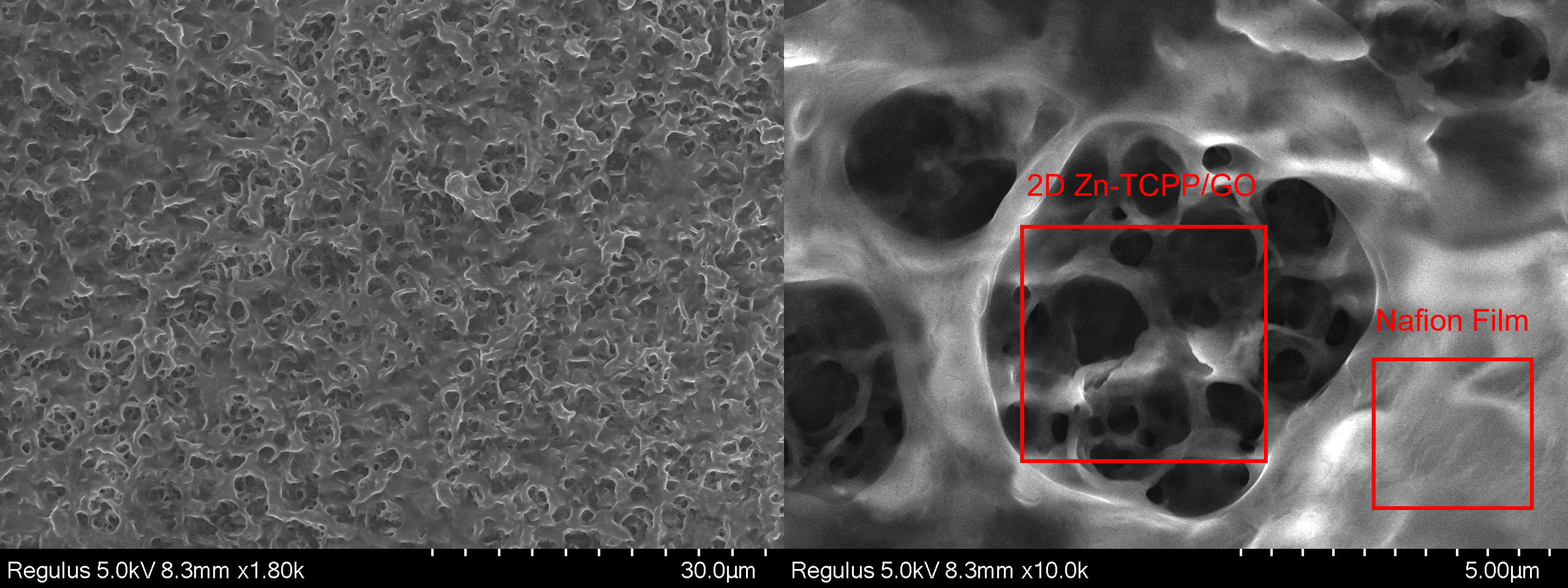


**Figure S19.** SEM images of Zn-TCPP/GO on the electrode surface. The outer thin film is derived from Nafion in the ink prepared for the sensing test.


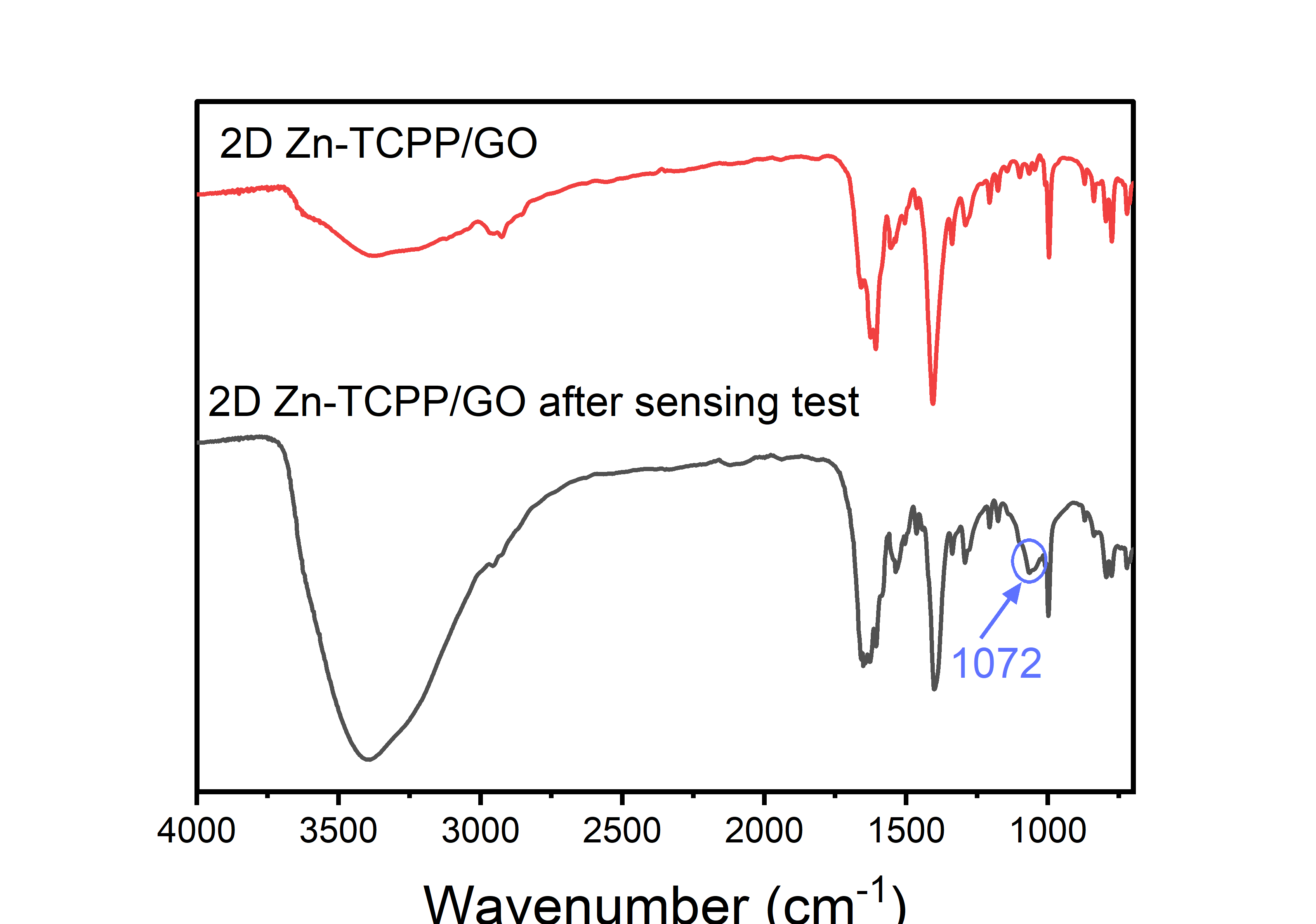


**Figure S20.** FT-IR spectra of 2D Zn-TCPP/GO before and after sensing test. The new peak emerging at 1072 cm^-1^ may be attributed to the adsorption of phosphate ions from the solution onto the surface of Zn-TCPP.

**
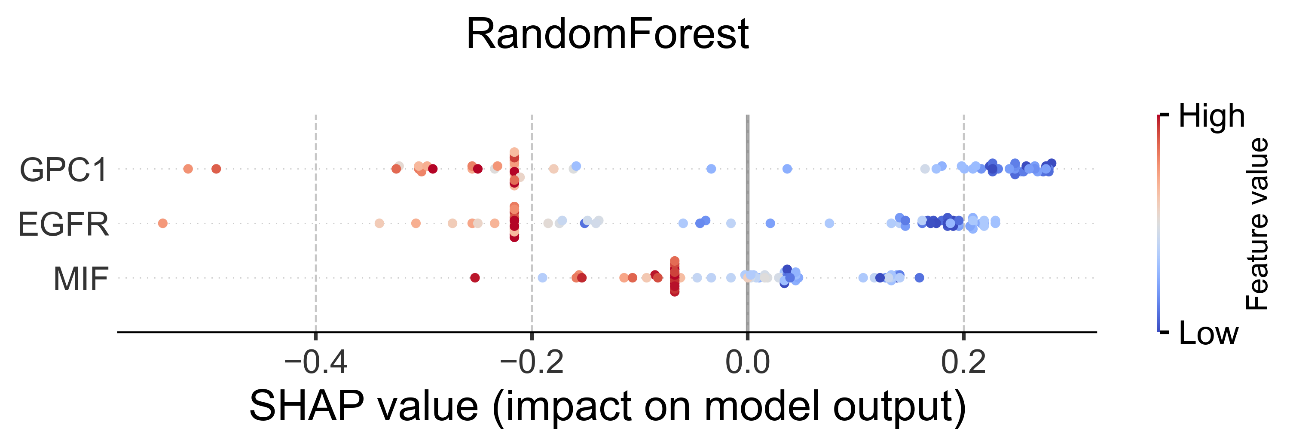
**

**Figure S21.** SHAP value analysis chart for the contribution of three biomarkers combined in the RF model.


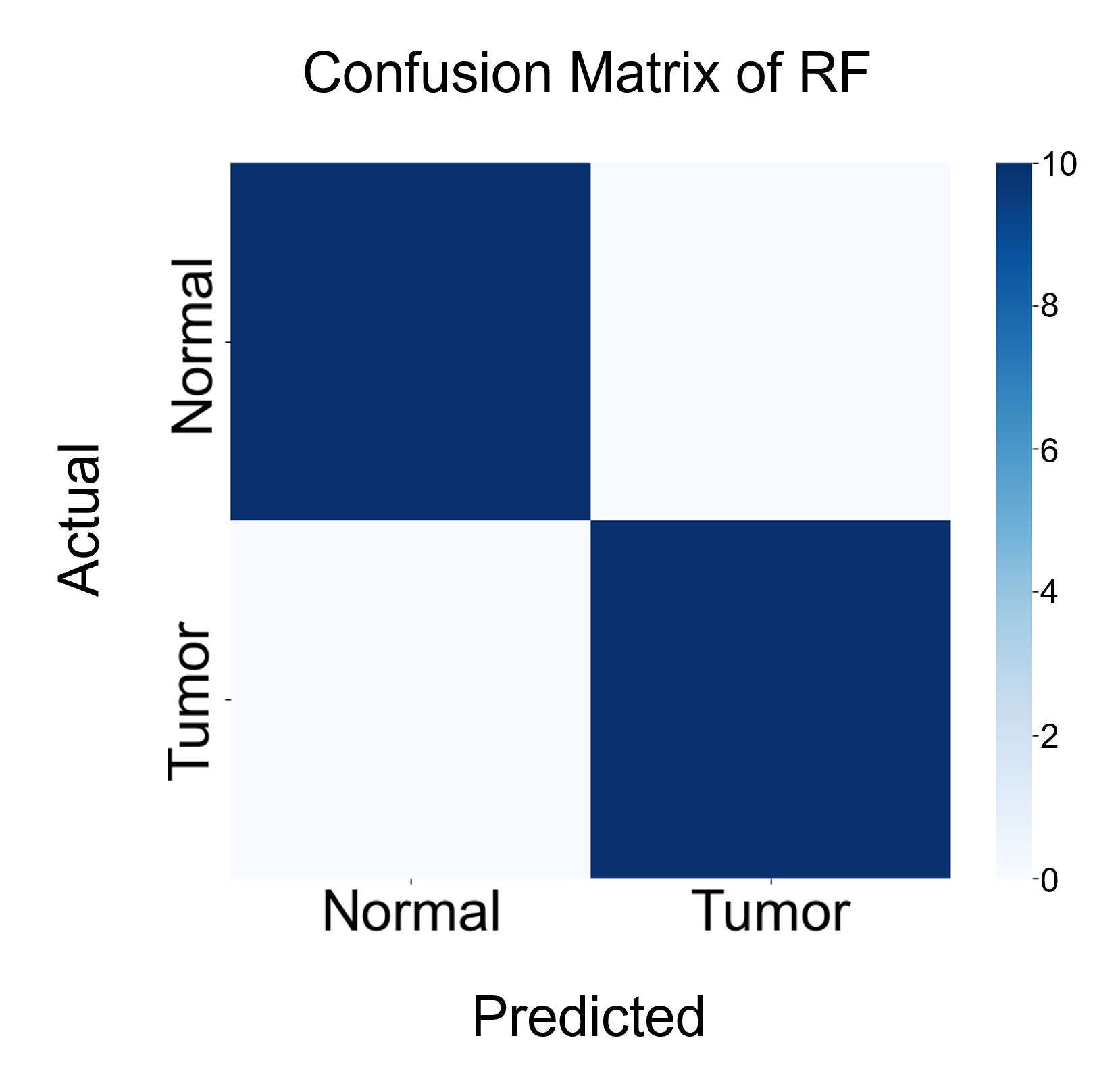


**Figure S22.** Confusion matrix of the RF model. The confusion matrix was used to evaluate the performance of a classification model by showing the comparison between actual classes and predicted classes, helping to analyze the classification accuracy.


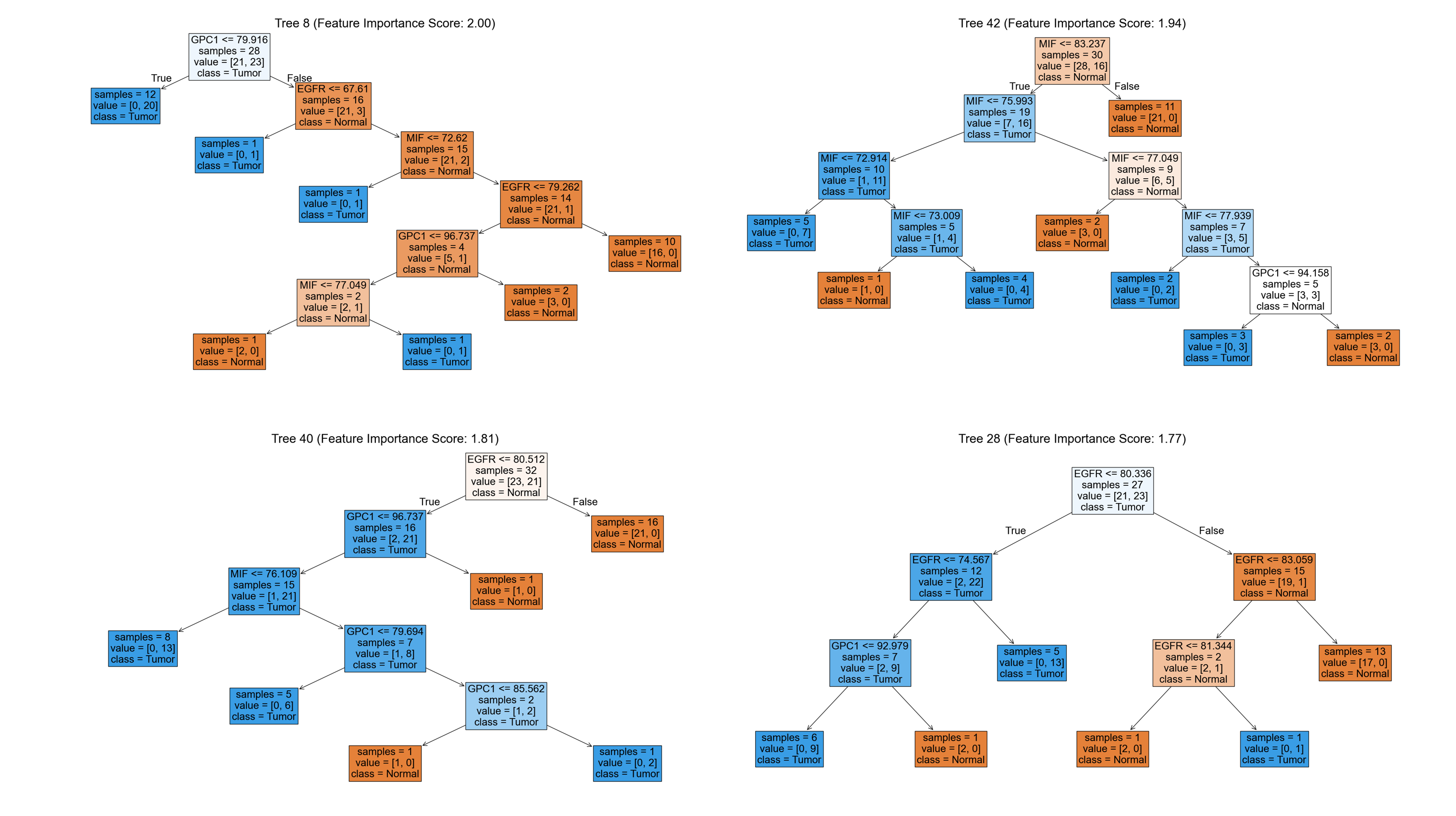


**Figure S23.** Four representative trees selected based on feature contributions in the RF model.

**Supplementary Tables**

**Table S1.** Structural parameters extracted from Zn K-edge EXAFS for 2D Zn-TCPP and bulk Zn-TCPP.

| Sample | Path | CN | R (Å) | σ^2^ | ΔE_0_ (eV) | (S_0_^2^) | R factor |
| --- | --- | --- | --- | --- | --- | --- | --- |
| Bulk Zn-TCPP | Zn-N | 3.9±0.2 | 1.94 | 0.005 | 3.9±0.5 | 0.7641 | 1.04% |
| 2D Zn-TCPP | Zn-N | 3.4±0.2 | 1.93 | 0.003 | 2.7±0.7 | 0.7641 | 1.01% |

CN is the coordination number; R is bonding distance; σ^2^ is Debye-Waller factor; ΔE_0_ is energy shift; S_0_^2^ is the amplitude reduction factor. Fitting range: 2.0 ≤ k ≤ 10.0 Å^-1^, 1.0 ≤ R ≤ 2.0 Å.

**Table S2.** The theoretical isoelectric points and hydrophilicity index of amino acids in peptides.

|  | Glutamic Acid (E) | L-arginine (R) | Histidine (H) | Proline (P) | Glycine (G) | Cysteine (C) |
| --- | --- | --- | --- | --- | --- | --- |
| Isoelectric Point | 3.22 | 10.76 | 7.59 | 6.30 | 5.97 | 5.02 |
| Hydrophilicity Index | -3.5 | -4.5 | -3.2 | -1.6 | -0.4 | 2.5 |

**Table S3.** The theoretical isoelectric points and hydrophilicity index of different peptides.

|  | Theoretical Isoelectric Point | Hydrophilicity Index |
| --- | --- | --- |
| EREREREGGGGC | 4.73 | -2.22 |
| EREREREPPPPC | 4.73 | -2.62 |
| HERHERHERGGGGC | 7.41 | -2.34 |
| HERHERHERPPPPC | 7.41 | -2.68 |

**Table S4.** Equations and parameters of the corresponding dose-dependent response to EGFR in DPV testing.

|  | 5-100 pg/mL | 500 pg/mL-1000 ng/mL |
| --- | --- | --- |
| equation | y = Intercept + B1*x^1 + B2*x^2 + B3*x^3 | |
| Intercept | 161.8115 | 354.0896 |
| B1 | -4.3326 | -178.5079 |
| B2 | -6.8549 | 42.6275 |
| B3 | 0.2281 | -3.5197 |
| Adjusted R² | 0.9993 | 0.9919 |

**Table S5.** Equations and parameters of the corresponding dose-dependent response to GPC-1 in DPV testing.

|  | 5-100 pg/mL | 500 pg/mL-1000 ng/mL |
| --- | --- | --- |
| equation | y = Intercept + B1*x^1 + B2*x^2 + B3*x^3 | |
| Intercept | 170.1371 | 286.6907 |
| B1 | -12.8567 | -122.7769 |
| B2 | 4.4246 | 29.9007 |
| B3 | -3.7690 | -2.6318 |
| Adjusted R² | 0.9911 | 0.9909 |

**Table S6.** Equations and parameters of the corresponding dose-dependent response to MIF in DPV testing.

|  | 5-100 pg/mL | 500 pg/mL-1000 ng/mL |
| --- | --- | --- |
| equation | y = Intercept + B1*x^1 + B2*x^2 + B3*x^3 | |
| Intercept | 152.5142 | 158.7995 |
| B1 | -8.4191 | -26.0684 |
| B2 | 2.4800 | 4.6312 |
| B3 | -3.0703 | -0.4245 |
| Adjusted R² | 0.9986 | 0.9974 |

**Table S7.** Clinical information collected from cancer patients.

| CEA (ng/mL)  Reference Range (<4.7) | 0.93 | 24.80↑ | 20.50↑ | 18.50↑ | 56.50↑ | 9.00↑ | 16.40↑ | 6.65↑ | 4.11 | 1.36 | 4.88↑ |
| --- | --- | --- | --- | --- | --- | --- | --- | --- | --- | --- | --- |
| CA242 (U/mL)  Reference Range (<20) | —— | —— | —— | 88.10↑ | 1000.00↑ | —— | —— | 10.20 | 9.46 | 11.80 | 17.30 |
| CA19-9 (U/mL)  Reference Range (≤37) | 438.00↑ | 573.00↑ | 680.00↑ | 158.00↑ | 1000.00↑ | 200.00↑ | 66.10↑ | 72.50↑ | 41.90↑ | —— | 132.00↑ |
| Comorbidity | Secondary malignant tumors of the liver, secondary malignant tumors of the abdominal cavity | —— | —— | —— | —— | Hypertension, renal insufficiency, moderate anemia | Severe anemia, anuria, shock, arrhythmia | —— | —— | —— | Diabetes, chronic gastritis, hypertension, coronary heart disease |
| Complication | —— | —— | Pulmonary infection | Obstructive jaundice | —— | —— | Ascites, hypoproteinemia, peritoneal effusion, infection, gastrointestinal obstruction | —— | —— | —— | —— |
| Clinical Diagnosis Result | Pancreatic malignant tumor | Pancreatic malignant tumor | Pancreatic malignant tumor | Pancreatic malignant tumor | Pancreatic malignant tumor | Pancreatic malignant tumor | Pancreatic malignant tumor | Pancreatic malignant tumor | Pancreatic malignant tumor | Pancreatic malignant tumor | Pancreatic malignant tumor |
| Age | 65 | 72 | 71 | 77 | 51 | 49 | 73 | 74 | 71 | 30 | 68 |
| Gender | Man | Man | Woman | Woman | Woman | Man | woman | Man | Woman | Woman | Man |
| No. | 1 | 2 | 3 | 4 | 5 | 6 | 7 | 8 | 9 | 10 | 11 |
| CEA (ng/mL)  Reference Range (<4.7) | 1.27 | 8.7↑ | 18.44↑ | —— | —— | 13.70↑ | 2.38 | 2.71 | 1.19 | 2.20 | —— |
| CA242 (U/mL)  Reference Range (<20) | 1.23 | 12.15 | 66.20↑ | —— | 31.70↑ | 7.80 | —— | —— | 3.00 | 6.68 | —— |
| CA19-9 (U/mL)  Reference Range (≤37) | 39.7↑ | 42.10↑ | 62.50↑ | 72.6↑ | 59.70↑ | 43.80↑ | 42.00↑ | 54.10↑ | 7.25 | 52.00↑ | 78.20↑ |
| Comorbidity | Chronic gastritis, Sjogren's syndrome | —— | —— | —— | —— | —— | —— | Hypertension | —— | Asthma, cholecystitis, bradycardia | —— |
| Complication | Yellowing of the skin and sclera | —— | Yellowing of the skin and sclera | Yellowing of the skin and sclera | Nausea and weakness | Vomiting and discomfort | Abdominal pain | There is a dull pain and discomfort in the upper right abdomen | Repeated abdominal pain | —— | —— |
| Clinical Diagnosis Result | Pancreatic malignant tumor | Pancreatic malignant tumor | Pancreatic malignant tumor | Pancreatic malignant tumor | Pancreatic malignant tumor | Pancreatic malignant tumor | Pancreatic malignant tumor | Pancreatic malignant tumor | Pancreatic malignant tumor | Pancreatic malignant tumor | Pancreatic malignant tumor |
| Age | 59 | 22 | 57 | 59 | 78 | 62 | 67 | 54 | 49 | 84 | 30 |
| Gender | Woman | Woman | Man | Man | Man | Woman | Man | Man | Woman | Man | Woman |
| No. | 12 | 13 | 14 | 15 | 16 | 17 | 18 | 19 | 20 | 21 | 22 |
| CEA (ng/mL)  Reference Range (<4.7) | 9.86↑ | 1.17 | 1.45 | 0.8 | 2.68 | 7.33↑ | 9.63↑ | 2.75 | 1.78 | 1.23 | —— |
| CA242 (U/mL)  Reference Range (<20) | 200.00↑ | 2 | 54.90↑ | 3.54 | 200.00↑ | 115.00↑ | 3.03 | 3.58 | 21.40↑ | 6.08 | 3.33 |
| CA19-9 (U/mL)  Reference Range (≤37) | 1000.00↑ | 5.58 | 72.70↑ | 5.16 | 930.00↑ | 449.00↑ | 45.40↑ | 7.03 | 68.50↑ | 12.70 | 12.90 |
| Comorbidity | Hypertension, diabetes | Diabetes | —— | —— | —— | Hypertension, thyroid surgery, rectal tumor | Mild anemia, hypokalemia, hypocalcemia | —— | —— | Coronary heart disease, chronic renal insufficiency | —— |
| Complication | —— | —— | —— | —— | —— | Abdominal distension | Yellow staining of the skin and sclera, incomplete intestinal obstruction, malignant ascites | —— | —— | Repeated abdominal pain | —— |
| Clinical Diagnosis Result | Pancreatic malignant tumor | Pancreatic malignant tumor | Pancreatic malignant tumor | Pancreatic malignant tumor | Pancreatic malignant tumor | Pancreatic malignant tumor | Pancreatic malignant tumor | Pancreatic malignant tumor | Pancreatic malignant tumor | Pancreatic malignant tumor | Acute pancreatitis |
| Age | 66 | 53 | 15 | 58 | 67 | 76 | 74 | 52 | 33 | 62 | 42 |
| Gender | Man | Woman | Woman | Woman | Woman | Man | Man | Man | Man | Man | Woman |
| No. | 23 | 24 | 25 | 26 | 27 | 28 | 29 | 30 | 31 | 32 | 33 |
| CEA (ng/mL)  Reference Range (<4.7) | 3.20 | 4.10 | 2.24 | 4.40 | 3.30 | 1.79 | 1.27 | 3.30 | 0.80 | 1.27 | 1.95 |
| CA242 (U/mL)  Reference Range (<20) | —— | —— | —— | —— | —— | 3.44 | 1.23 | —— | —— | —— | —— |
| CA19-9 (U/mL)  Reference Range (≤37) | 22.20 | 12.80 | 26.90 | 9.80 | 18.70 | 8.89 | 38.70↑ | 18.70 | 8.60 | 39.70↑ | 40.80↑ |
| Comorbidity | —— | —— | —— | —— | —— | —— | The history of coronary heart disease is over ten years | Premature beats, benign prostatic hyperplasia, coronary heart disease, cerebral atherosclerosis, hypertension | diabetes | —— | Diabetes |
| Complication | Persistent dull pain in the upper abdomen | Persistent dull pain in the upper abdomen | Persistent dull pain in the upper abdomen | Persistent dull pain in the upper abdomen | Persistent dull pain in the upper abdomen | —— | —— | Persistent dull pain in the upper abdomen | Persistent dull pain in the upper abdomen | —— | —— |
| Clinical Diagnosis Result | Acute pancreatitis | Acute pancreatitis | Acute pancreatitis | Acute pancreatitis | Acute pancreatitis | Acute pancreatitis | Acute pancreatitis | Acute pancreatitis | Acute pancreatitis | Acute pancreatitis | Acute pancreatitis |
| Age | 47 | 35 | 91 | 71 | 87 | 38 | 68 | 87 | 33 | 68 | 44 |
| Gender | Man | Man | Woman | Woman | Man | Man | Man | Man | Woman | Man | Man |
| No. | 34 | 35 | 36 | 37 | 38 | 39 | 40 | 41 | 42 | 43 | 44 |

The results obtained through clinical chemiluminescence immunoassay (CLIA) indicate that the sensitivity and specificity of biomarkers associated with pancreatic cancer, such as CA19-9, CA24-2, and CEA, are as follows: CA19-9 (with a sensitivity of 0.84 and a specificity of 0.75), CA24-2 (with a sensitivity of 0.39 and a specificity of 1.00), and CEA (with a sensitivity of 0.48 and a specificity of 1.00).

**Table S8.** The comparative analysis of various detection methods or technologies.

| LOD  (pg/mL) | Total Analysis Duration | Accuracy/  Sensitivity/Specificity | Sample Pretreatment Required | Ref |
| --- | --- | --- | --- | --- |
| 10^5^ | 45 min | 100% | YES | [3] |
| 4.50×10^−2^ | 2 h 15 min | 92.9% | YES | [4] |
| 6.93×10^−6^ | 12 h | 94% | YES | [5] |
| 20 | 40 min | 87.5 % | YES | [6] |
| 5 | 30 min | 100 % | NO | This work |

**Supplementary References**

[1] L. Tao, X. Wang, F. Wu, B. Wang, C. Gao, X. Gao, *Separation and Purification Technology* **2022**, *296*, 121309.

[2] J. Cui, J. Luo, B. Peng, X. Zhang, Y. Zhang, Y. Wang, Y. Qin, H. Zheng, X. Shu, Y. Wu, *Nanoscale* **2015**, *8*, 770.

[3] T. Yin, L. Xu, B. Gil, N. Merali, M. S. Sokolikova, D. C. A. Gaboriau, D. S. K. Liu, A. N. Muhammad Mustafa, S. Alodan, M. Chen, O. Txoperena, M. Arrastua, J. M. Gomez, N. Ontoso, M. Elicegui, E. Torres, D. Li, C. Mattevi, A. E. Frampton, L. R. Jiao, S. Ramadan, N. Klein, *ACS Nano* **2023**, *17*, 14619.

[4] J. He, J. Long, C. Zhai, J. Xu, K. Bao, W. Su, L. Jiang, G. Shen, X. Ding, *Anal. Chem.* **2024**, *96*, 6618.

[5] A. N. Masterson, N. N. Chowdhury, Y. Fang, M. T. Yip-Schneider, S. Hati, P. Gupta, S. Cao, H. Wu, C. M. Schmidt, M. L. Fishel, R. Sardar, *ACS Sens.* **2023**, *8*, 1085.

[6] X. Duan, L. Zhao, H. Dong, W. Zhao, S. Liu, G. Sui, *ACS Sens.* **2019**, *4*, 2952.
